# Microsporidia-nematode associations in methane seeps reveal basal fungal parasitism in the deep sea

**DOI:** 10.3389/fmicb.2014.00043

**Published:** 2014-02-10

**Authors:** Amir Sapir, Adler R. Dillman, Stephanie A. Connon, Benjamin M. Grupe, Jeroen Ingels, Manuel Mundo-Ocampo, Lisa A. Levin, James G. Baldwin, Victoria J. Orphan, Paul W. Sternberg

**Affiliations:** ^1^Howard Hughes Medical Institute and Division of Biology, California Institute of TechnologyPasadena, CA, USA; ^2^Division of Geological and Planetary Sciences, California Institute of TechnologyPasadena, CA, USA; ^3^Integrative Oceanography Division, Center for Marine Biodiversity and Conservation, Scripps Institution of OceanographyLa Jolla, CA, USA; ^4^Marine Life Support Systems, Plymouth Marine Laboratory, Prospect PlaceUK; ^5^Department of Agricultural Biotecnology, Nematology Laboratory, CIIDIR-IPN Unidad SinaloaSinaloa, Mexico; ^6^Department of Nematology, University of CaliforniaRiverside, CA, USA

**Keywords:** deep-sea methane seeps, nematodes hosts, deep-sea microsporidia parasitism, muscle decomposition, basal fungi in the deep sea

## Abstract

The deep sea is Earth's largest habitat but little is known about the nature of deep-sea parasitism. In contrast to a few characterized cases of bacterial and protistan parasites, the existence and biological significance of deep-sea parasitic fungi is yet to be understood. Here we report the discovery of a fungus-related parasitic microsporidium, *Nematocenator marisprofundi* n. gen. n. sp. that infects benthic nematodes at methane seeps on the Pacific Ocean floor. This infection is species-specific and has been temporally and spatially stable over 2 years of sampling, indicating an ecologically consistent host-parasite interaction. A high distribution of spores in the reproductive tracts of infected males and females and their absence from host nematodes' intestines suggests a sexual transmission strategy in contrast to the fecal-oral transmission of most microsporidia. *N. marisprofundi* targets the host's body wall muscles causing cell lysis, and in severe infection even muscle filament degradation. Phylogenetic analyses placed *N. marisprofundi* in a novel and basal clade not closely related to any described microsporidia clade, suggesting either that microsporidia-nematode parasitism occurred early in microsporidia evolution or that host specialization occurred late in an ancient deep-sea microsporidian lineage. Our findings reveal that methane seeps support complex ecosystems involving interkingdom interactions between bacteria, nematodes, and parasitic fungi and that microsporidia parasitism exists also in the deep-sea biosphere.

## Introduction

A major component of life in the deep sea relies on chemosynthetic energy emitted at hydrothermal vents and cold methane seeps, where prokaryotes consume sulfide or methane for primary production supporting oasis-like ecosystems rich in biomass (Sibuet and Olu, 1998; Martin et al., 2008). In methane seeps, methane oxidation coupled with sulfate reduction is mediated by anaerobic consortia of archaea and bacteria (Knittel and Boetius, 2009). The level of methane, a potent green house gas released to the water column from seeps, is determined in part by the growth and activity of methane-fueled autotrophs and the eukaryotes that graze upon them (Thurber et al., 2012). In contrast to the well-established interactions between prokaryotes and animals at vents and seeps, little is known about the nature, abundance, life strategies, and ecological significance of eukaryotic microorganisms living in chemosynthetic ecosystems (Takishita et al., 2007).

Due to sampling challenges, limited access, and difficulties of growing and maintaining deep-sea organisms in the lab, our knowledge of deep-sea fungal life is limited and relies primarily on the study of a few cultureable species and on culture-free approaches such as metagenomic sampling. Fungi recovered from the deep sea include free-living yeasts that were reported to dominate fungal diversity in the deep oceans (Bass et al., 2007) (Nagahama et al., 2011) and various filamentous species (Burgaud et al., 2009; Thaler et al., 2012). Although some deep-sea fungi are closely related to known terrestrial species, recent studies of fungal diversity at hydrothermal vents (Burgaud et al., 2011) and cold seep habitats (Thaler et al., 2012) resulted in the discovery of novel fungal clades suggested to be endemic to the deep sea. Even though the majority of fungi sampled from chemoautotrophic environments are thought to be free-living, a few yeast species of the genus *Rhodotorulahave* were found in association with deep-sea tubeworms and clams (Nagahama et al., 2003). The possible effect of these free-living and animal-associated fungi in deep-sea benthic ecosystems is yet to be characterized. In contrast, the pathogenic effect of a mussel-infecting parasitic fungus found in a Fiji Basin hydrothermal vent (Van Dover et al., 2007) demonstrates the significant role parasitic fungi can play in benthic ecosystem dynamics. Sequence-based fungal surveys of cold seeps (Nagahama et al., 2011) revealed that the most frequently recovered clones were of early diverging basal fungi, including two species of *Rozella*, an endoparasitic genus that, along with microsporidia are considered to be the earliest branching taxa of sequenced fungi (James et al., 2006; Capella-Gutierrez et al., 2012). The details of *Rozella* parasitism and its biological significance in the context of methane seep ecosystems are yet to be understood.

Microsporidia is a phylum of fungal obligate intracellular eukaryotic parasites that infect a wide range of hosts from most metazoan phyla. Understanding microsporidia biology is of medical and agricultural interests due to the mortality they cause among immunocompromised humans, their use in biocontrol against agriculturally damaging insects (Lacey et al., 2001), and the proposed role of microsporidia infection in the colony collapse disorder of honeybees reviewed in Texier et al. (2010). Many species of microsporidia are known to infect the host's intestine, including the human pathogens that can cause severe and persistent diarrhea in immunocompromised patients (Lewthwaite et al., 2005). Microsporidia cycle between intracellular phases where a cell-wall deficient meront replicates and differentiates to form sporonts. The sporonts differentiate further, undergoing sporulation (sporogony plus sporogenesis) to eventually form spores (Keeling and Fast, 2002). The spores can either be released into the environment to be ingested by a new host (horizontal transmission) or passed from parent to offspring via the eggs (vertical transmission). In most studied cases, spore entry into a naïve host and association with target cells leads to the activation of an infection apparatus—the polar filament that in a non-activated state remains coiled inside the spore. The filament, when extruded from the spore to form the polar tube, pierces the host cell membrane, injecting nuclei and sporoplasm into the host cell's cytoplasm to initiate a new infection cycle (Xu and Weiss, 2005). During their evolution toward obligate parasitism, the mitochondria of microsporidia have evolved into mitosomes—mitochondrially derived organelles that have retained some metabolic function but lost the ability to produce energy via oxidative phosphorylation (Katinka et al., 2001; Burri et al., 2006; Tsaousis et al., 2008). Thus, microsporidia bioenergetics relies either on anaerobic substrate-level metabolism, on ATP supply from the host, or both. Furthermore, whole genome sequencing of selected microsporidia species revealed an extreme degree of genome compaction and reduction with some species harboring the smallest genomes among known eukaryotes (Keeling et al., 2005; Cuomo et al., 2012). So far, only one case of microsporidia infection in the deep sea is reported but is actually carried out by the metazoan parasite Myxosporea (Dubina and Isakov, 1976). Although little is known about microsporidia in the deep sea, microsporidia were recently shown by ultrastructural and molecular approaches to be genuine parasites of hosts in various marine habitats including shallow water fish and nematodes in the low tide zone (Abdel-Ghaffar et al., 2011; Ardila-Garcia and Fast, 2012).

Nematoda is perhaps the most abundant and versatile animal phylum on earth with adaptations to a vast spectrum of habitats including extreme environments such as the frozen Antarctic Dry Valleys (Adams et al., 2007) and even the Earth's crust (Borgonie et al., 2011). Despite their ecological diversity, nematodes all have a non-segmented body comprised of an endodermal alimentary system, a mesodermal muscle and reproductive system, and an ectodermal hypodermis covered by a thick extracellular cuticle. The nematode cuticle restricts interaction of nematodes with their environment mainly to orifices such as the mouth, anus, some sensory organs (e.g., amphids), and the vulva and cloaca of females and males respectively. The cuticle and body orifices are the sites where nematode-associated microorganisms can be found, often as food sources or associated symbionts. For example, the ectosymbiosis between marine nematodes and cuticle-associated sulfur-oxidizing bacteria is well-established (Polz et al., 1992; Bulgheresi et al., 2011), and sometimes even nematode-bacteria endosymbioses are established (Musat et al., 2007; Tchesunov et al., 2012). Bacterivorous nematodes are probably the most abundant meiofauna (animals ranging in size between 32 μm and 1 mm) living in many deep-sea habitats (Ingels et al., 2011; Pawlowski et al., 2011) including hydrothermal vents and methane seeps, where nematode diets include chemoautotrophic bacteria (Vanreusel et al., 2010a,b). Nematode abundance in the deep oceans makes them potential hosts for endoparasitic microorganisms. However, to our knowledge this type of parasitism has not been reported yet.

Surveying for possible microorganism-nematode interactions in the deep sea, we found microsporidia parasitism to be a novel, genuine, and temporally stable life strategy at Hydrate Ridge methane seeps in the Pacific Ocean. Phylogenetic analyses concordantly show that the microsporidia we describe constitute a novel, basal clade of deep-sea microsporida. Our study suggests that a separate branch of basal microsporidians has evolved in the deep sea and highlights the potential effect of microsporidia parasitism on deep-sea chemoautotrophic ecosystems.

## Results

### Microsporidia parasitism at hydrate ridge methane seeps

**Figure 1:**
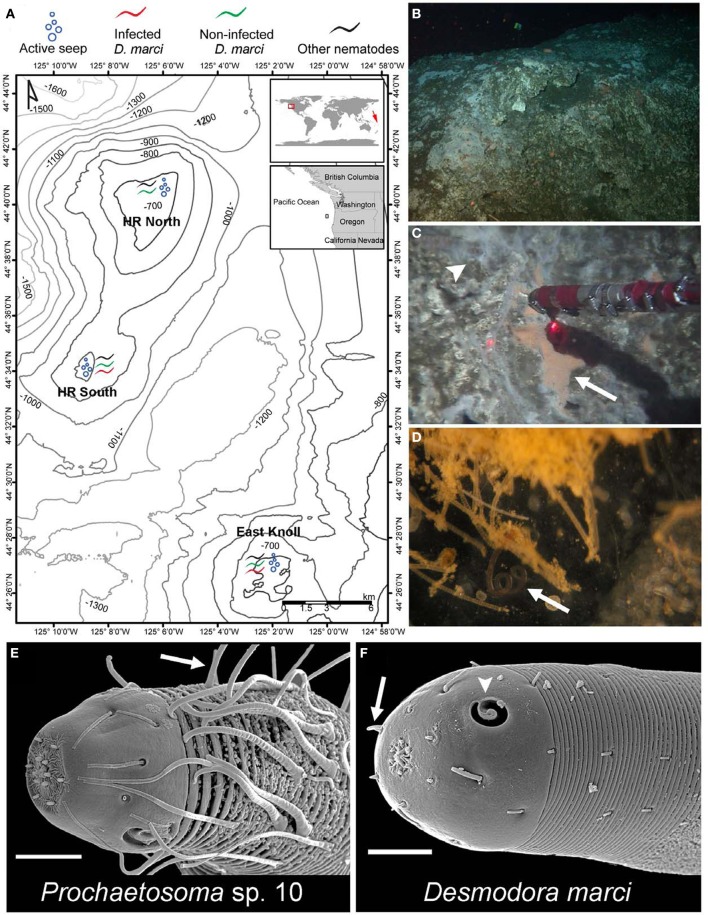
**Hydrate Ridge methane seeps are inhabited by *Desmodora* and *Prochaetosoma* nematodes. (A)** A map of Hydrate Ridge with the 2010 and 2011 sampling sites and location where infected and non-infected *D. marci* worms were found. On the global map, a red square marks HR where we found infected *D. marci* nematodes. A red arrow marks the location of the Lau Basin hydrothermal vent systems where *D. marci* was previously reported (Verschelde et al., 1998). **(B)** *In situ* photo of a typical HR carbonate rock. **(C)** Orange bacterial mat (arrow) on a carbonate rock within an active methane seep (arrowhead) collected by hydraulic suction. The distance between laser points is 10 cm. **(D)** A *D. marci* worm (arrow) associated with the bacterial mat. The image was taken on the ship after sampling. **(E)** SEM micrograph of the head of *Prochaetosoma* sp. 10 with typical long cephalic adhesion tubes connected to adhesive glands, presumeably used for locomotion (arrow). **(F)** SEM micrograph of the head of *D. marci* with two cryptospiral amphid sensory organs (1.2 turns, arrowheads) and four submedian cephalic papilla (arrow). Scale bars are 10 μm **(E,F)**.

**Figure 2:**
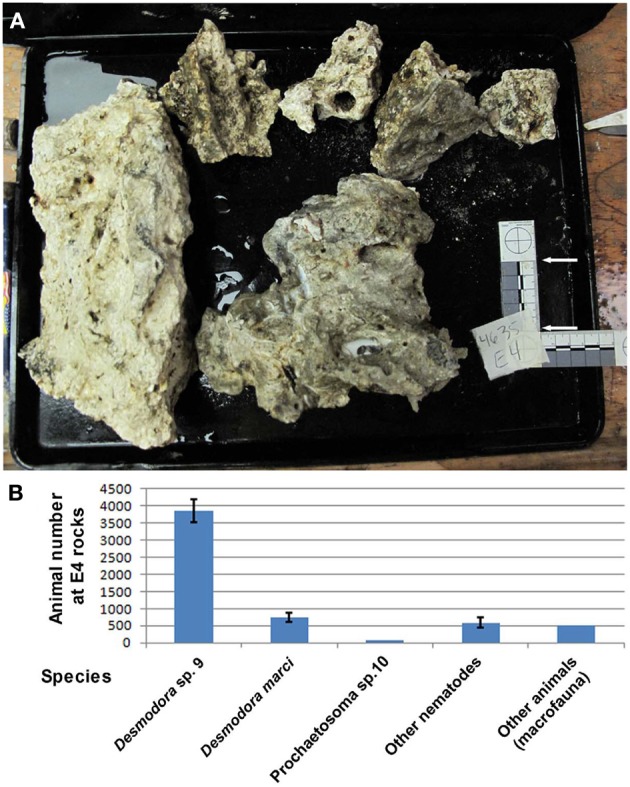
***Desmodora* nematodes dominate Hydrate Ridge carbonate rock ecosystems**. **(A)** Hydrate Ridge South E4 carbonate rocks that were sampled and processed on the ship in August 2010. The combined surface area of these rocks is 0.1615 m^2^. **(B)** Animal densities at Hydrate Ridge south E4 rock calculated as individuals per combined E4 rock. Nematode numbers represent the average of three samples 1/120th of the total fraction recovered from the rocks. “Other animals” are the total number of meiofauna and macrofauna (mainly Polychaeta) found in E4 rock. *Desmodora* and *Prochaetosoma* worms are 83.6% of the total meio and macrofauna animals living on E4 rock. Bars represent standard errors.

**Figure 3:**
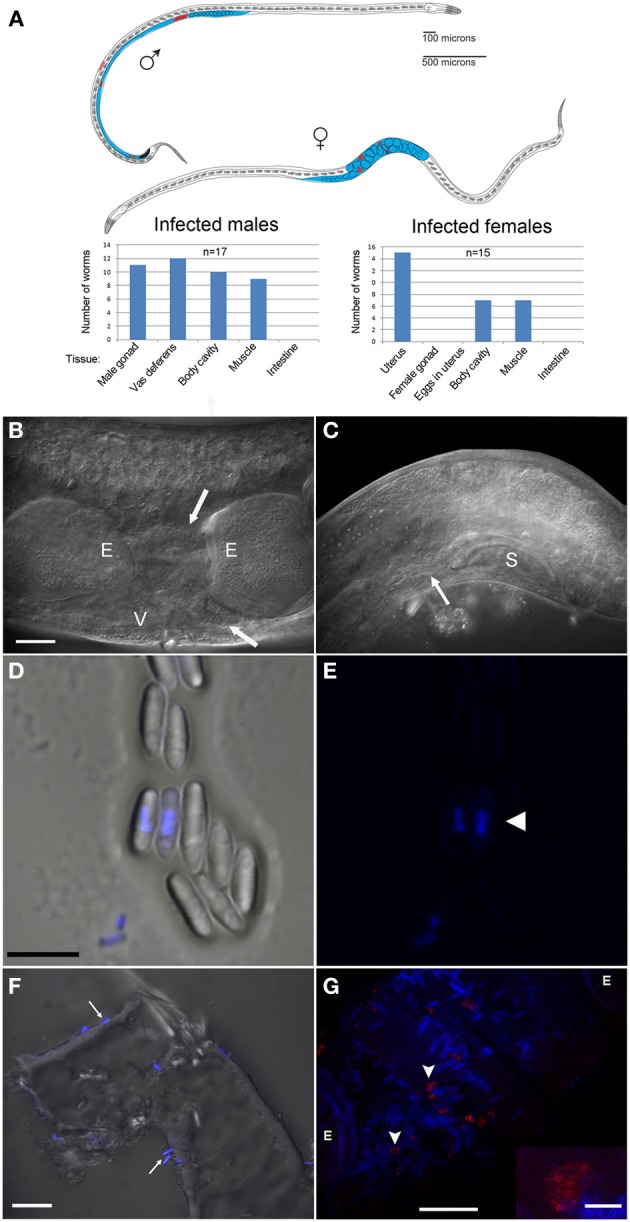
***N. marisprofundi* is associated with *D. marci* reproductive organs. (A)** A diagram of infected *D. marci* male and female. Gonads, male vas deferens, and female uterus are labeled in blue. Microsporidia spores are labeled in red. The bar graphs show the tissue-specific patterns of spore distribution in males and females, numbers are not exclusive. **(B)** Nomarski DIC of a moderately infected female. E, eggs; V, vulva; arrow, microsporidia spores. **(C)** *N. marisprofundi* infection of male reproductive organs. Microsporidia spores are distributed at the end of the vas deferens (arrow) and at the dorsal posterior side; S, male spicule; magnification as in B. **(D)** *N. marisprofundi* spores dissected from a female genital tract and stained by 4′,6-diamidino-2-phenylindole (DAPI). **(E)** DAPI staining (blue) revealed nucleus-like structure inside the spores (arrowhead) magnification as in **(D)**. **(F)** Calcofluor white staining (blue) of *N. marisprofundi* spores (arrows) attached to a dissected worm cuticle. **(G)** *In situ* hybridization with *N. marisprofundi* SSU rRNA probe (red) and DAPI staining (blue) of an infected female fixed by paraformaldehyde to detect specifically the vegetative stages (see Materials and Methods section in Supporting Information). Vegetative stages (arrowheads) are positioned in the vicinity of spores. E developing embryo. Scale bars are 20 μm **(B,F,G)**; 5 μ m **(D)**; insert 2.5 μm **(G)**.

While surveying for possible symbioses between microorganisms and deep-sea nematodes, we found abundant nematode assemblages associated with carbonate rocks and sulfide-oxidizing bacterial mats at Hydrate Ridge (HR) methane seeps, 85 km off the coast of Oregon at 587–810 m water depth (Figures 1A, S1). In this low oxygen environment (minimum O_2_ concentration of about 0.25 ml/L) nematodes comprise 80% or more of all meiofauna (Guilini et al., 2012). We discovered that the second most abundant nematode species collected on carbonate rocks near an active seepage (Figure 2) is associated with orange pigmented sulfide oxidizing bacterial mats (Figure 1C) and harbors rod-like microbes in its reproductive system (Figures 3A–C). Based on small ribosomal subunit (SSU) rDNA analysis we identified the nematode as a *Desmodora* species (Figure S4). Detailed morphological study indicated that the nematode is *Desmodora marci* (Superfamily Desmodoroidea, family Desmodoridae, Figures 1F, S2–S3) a species originally described from hydrothermal vents in the West Pacific Lau Basin near Fiji (Hine Hina site) (Verschelde et al., 1998) and we refer to it as *D. marci* throughout. The original description of this species also mentions the presence of micro-organisms associated with the nematode cuticle. Morphologically-distinct rod-like microbes were also detected in another, closely related nematode from the HR assemblage that was identified as a *Prochaetosoma* species (superfamily Desmodoroidea, family Draconematidae) and we refer to it as *Prochaetosoma* sp. 10 (Figures 1E, S2–S3). The most abundant nematode was identified as another *Desmodora* species (affinity with *Desmodora alberti*), closely related to *D. marci*. We refer to this species as *Desmodora* sp. 9. *Desmodora alberti* was also originally described from hydrothermal vent habitats at the East Pacific Rise (Verschelde et al., 1998). This nematode showed no signs of infection by spore-forming microbes (*n* = 40).

Because the microbes exhibited a nucleus-like structure (Figures 3D,E; Movie S1), the possibility of fungal infection was examined by the use of general fungal primers and by Calcofluor White, a dye that stains the fungal cell wall. Although the lack of PCR product suggested that the well-characterized species of fungi are not involved, a positive spore-restrictive Calcofluor White staining indicated infection by a spore-forming fungus or protist (Figure 3F). Among fungus-related organisms, microsporidia were recently characterized as nematode parasites (Troemel et al., 2008; Ardila-Garcia and Fast, 2012). We used microsporidia-specific primers (Ghosh and Weiss, 2009) and found one rDNA SSU amplicon specific to infected *D. marci* that is similar by BLAST to known microsporidia SSU rDNA sequences. We name this microsporidium *Nematocenator marisprofundi* n. gen. n. sp., Latin for “nematode eater of the deep sea” and will refer to it as *N. marisprofundi* throughout (see formal genus and species descriptions in the microsporidia taxonomy section). We used the same microsporidia-specific primers on a few spores-harboring *Prochaetosoma* sp. 10 nematodes (five males and one female out of five adult males and three adult females analyzed) and found that these worms are infected with a species of microsporidia closely related to *N. marisprofundi* we named *Nematocenator* sp. 1 (Figure 6). As *Prochaetosoma* sp. 10 nematodes were hard to sort due to their small size and low abundance in samples (Figure 2), we focused on the *D. marci* and *N. marisprofundi* interaction.

### A genuine and temporarily stable microsporidia parasitism of live nematode hosts

**Figure 4:**
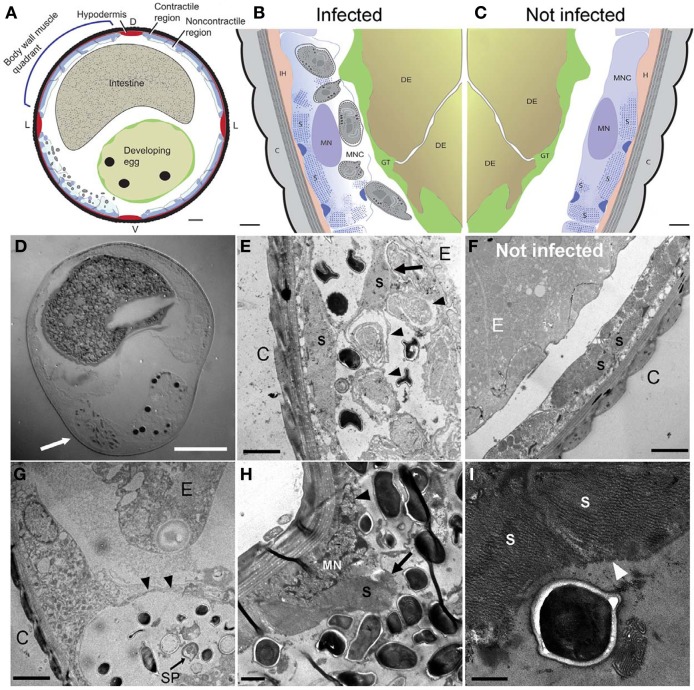
***N. marisprofundi* infection induces host muscle decomposition. (A–C)** Diagrams based on many TEM micrographs integrated to show body wall muscle decomposition by *N. marisprofundi*. C, cuticle; H, hypodermis; S, sarcomeres; MN, muscle nucleus; GT, genital tract, DE developing egg, MNC muscle non-contractile region. Microsporidia spores are represented by gray ovals. **(D)** A Nomarski DIC transverse section of an infected female harboring spores (arrow). **(E,F)** TEM micrographs of infected **(E)** and non-infected **(F)**, worms. C, cuticle; MFs, muscle filaments; E, developing egg. Developing spores (arrowheads) are localized near detached muscle filaments (arrow). **(G)** Sporoblast (SP) and spores in a cyst-like host tissue (arrowheads) C, cuticle; E, developing egg. **(H)** Muscle filaments (arrow) and nucleus (arrowhead) decomposition in a severely infected host. **(I)** Muscle filaments (arrowheads) consumed by *N. marisprofundi*. Scale bars are 20 μm **(D)**; 10 μm **(A)**; 1 μm **(B,C,H)**; 2 μm **(E–G)**; 0.5 μm **(I)**.

Because our samples were obtained from a deep-sea seep environment where microsporidia have not previously been reported, we rigorously tested whether the observed nematode-microsporidia interaction is both genuine and ecologically relevant using the following procedures: (i) Sampling at HR for two consecutive years we found that the same species of nematode host is infected with same species of microsporidia. This sampling demonstrates the spatially and temporally stable interaction of *N. marisprofundi* with its nematode host at HR methane seeps (Figure S1). (ii) Spores were detected in samples fixed immediately after sampling, precluding secondary infection during transport or in the lab. (iii) Live nematodes collected from HR that were kept alive at 4°C in semi-dysoxic conditions in the lab were also found to harbor spores, demonstrating microsporidan association within live hosts (*n* = 4.8; Figure 6; Movie S2). (iv) Lack of infection (*n* = 40) in the closely related nematode, *Desmodora* sp.9 (Figures 4, S2) demonstrates host species specificity of *N. marisprofundi*. Notably, a high level of host specificity is not uncommon among microsporidia (Porter et al., 2007). Taken together, these results indicate that HR active methane seeps are stable habitats of abundant bacterivorous nematode and their microsporidia parasites.

### Geographically wide spread parasitism at hydrate ridge seeps

To characterize better host-parasite interactions, we examined the spatial and temporal patterns of nematode-microsporidia association at the various methane seeps distributed along Hydrate Ridge. We sampled environments representing the range of ecosystems at HR North, South, and East Knoll, spanning deep seeps (700–800 water depth m) overlain by low oxygen waters and shallower seeps (500–600 m) where oxygen concentration is higher (Figures 1A, S1; Table S1). These environments include carbonate rocks and sediments with active methane emissions (active seeps) and inactive sites 30–300 m from the nearest active seepage. Host nematodes were abundant in carbonate samples from active seeps (about 20 worms/ml) but absent in samples from inactive seeps or sediments (Table S1), suggesting that sulfide oxidizing mats and carbonates from active seep areas are the preferred niches for the nematode host and parasite.

*D. marci* nematodes were associated with bacterial mats on carbonate rocks in all three regions of HR (Figure 1A), but in contrast to HR South and East Knoll, HR North worms were not infected (*n* = 141). To understand the dynamics of host-parasite distribution in the various ecological niches of HR we placed substrates (wood, rock, and bone) near inactive and active seeps for yearlong colonization experiments. Nematodes were extremely rare in substrates placed next to inactive seeps but recovered, in large numbers, from active seeps. At HR North we found uninfected *D. marci* to be the dominant species associated with carbonate rocks, whereas wood and bones were populated mainly by other nematode species (Table S1). In HR South seeps, however, *D. marci* worms were the dominant nematode species recovered from rock, wood, and bone substrates. Infected worms were found to be associated with all three substrates but the highest infection rate (as high as 63.33% of the examined animals) was of nematodes colonizing rocks (Table S1). Taken together, the finding of microsporidia in nematodes collected from HR South and East Knoll [about 15 km apart (Figure 1A)] demonstrates that microsporidia parasitism is a characteristic of HR ecosystems whereas colonization experiments demonstrate the potential of these parasites to spread into new ecological habitats by infected hosts.

### *N. marisprofundi* distribution within the host suggests a sexual transmission mechanism

Because of the extreme conditions at HR (e.g., low oxygen and high pressure, see Supporting Information) we could not sustainably culture HR nematodes and the microsporidia they harbor. Therefore, the dynamics of infection were deciphered based on positional and morphological analysis of microsporidia-infected nematodes that were preserved by shipboard fixation. To estimate the level of infection within the nematode population, we focused on two samples collected from the active seeps of HR South that were rich in *D. marci* nematodes. Either due to sampling bias or the nematode life cycles, *D. marci* juveniles were rare in HR samples precluding comprehensive analysis of infection in younger nematode stages. Over 90% of adult *D. marci* were alive at the time of fixation and 50–62% of male and female worms from both samples harbored microsporidia (*n* = 106; Table S1).

Many microsporidia, including the terrestrial nematode infecting species *Nematocida parisii* and *Nematocida* sp. 1, are known to be horizontally transmitted by fecal-oral infection cycles (Troemel et al., 2008). Surprisingly, despite the functional and morphological similarities of *C. elegans* and *D. marci* intestines, we have never observed microsporidia spores in the intestine of *D. marci* (*n* = 100), nor did we find evidence of gut malformations typical of microsporidia infections in *C. elegans* (Estes et al., 2011). Concurrently, free spores were neither detected in a survey of the samples most densely populated with infected *D. marci* (about 20 worms/ml) nor in other animals living in close proximity to the infected nematodes such as adult Copepoda, Polychaeta, and Mollusca (*n* = 35).

Some microsporidia species are also vertically transmitted by females to their offspring as spores inside developing eggs (Becnel et al., 1987) or by deposition of sporoplasms into developing oocytes via spores localized within ovaries (Terry et al., 1997). Sexual transmission has been hypothesized to occur in some species of microsporidia, but is not thought to be common or important in transmission, even in observed cases (Goertz and Hoch, 2011). In contrast to this paradigm, we have not detected spores in the female gonads or inside eggs (*n* = 94 eggs dissected from 12 heavily infected females) but rather in the uterus, between the eggs. Moreover, at least five infected *D. marci* males had spores in the vas deferens and cloaca (Figure 3C). The most compelling explanation of these observations is that *N. marisprofundi* is transmitted sexually, although we cannot rule out a vertical transmission as described for *Sporanauta perivermis*, a microsporidium recently found to infect nematodes in the low tide zone of Boundary Bay beach, Canada (Ardila-Garcia and Fast, 2012).

### *N. marsiprofundi* decomposes the host's body wall muscles

**Figure 5:**
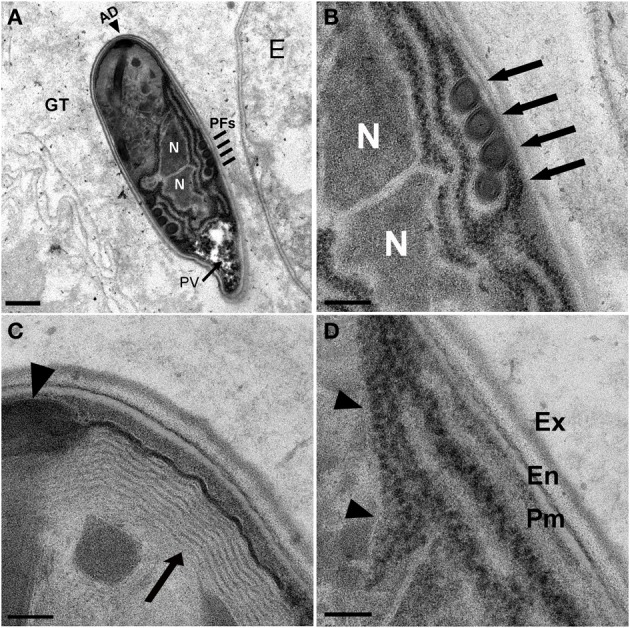
***N. marisprofundi* spore morphology. (A–D)** TEM micrographs of 70 nm longitudinal cross sections showing *N. marisprofundi* spore morphology. **(A)** Whole mature spore with a typical coils of the polar filaments. Coil number varies between three and five (*n* = 15 spores imaged), no spore dimorphism has been observed. PV, posterior vacuole (arrow); N, nucleus; PFs, four coils of the polar filaments (lines); AD, anterior disc (arrowhead), GT genital tract. **(B)** A close up of the central region showing the two nuclei (N) and four isofilar polar filament coils (arrows) **(C)** The lamellar polaroplast (arrow) underneath the anchoring disc (arrowhead) at the anterior end of the spore. **(D)** Exospore (Ex) Endospore (En), plasma membrane (Pm) at the spore periphery, and a row of ribosomes adjacent to a nucleus (arrowheads). Nomenclature of spore components is based on (Vavra and Larsson, 1999). Scale bars are 1 μm **(A)**; 200 nm **(B)**; 100 nm **(C,D)**.

Microsporidia have a complex intracellular life cycle restructuring organelles and other components of the infected host cell to support the parasite metabolism, development, and reproduction (Troemel et al., 2008). To understand the infection dynamics, we used *in situ* fluorescent hybridization (FISH) with *N. marisprofundi* SSU rRNA probes and Transmission Electron Microscopy (TEM). Fluorescent signal localization to the spore-containing areas (Figure 3G) demonstrated that the intracellular stages are co-localized with spores in the female host uterus or adjacent muscle cells. TEM analysis of many thin sections of three infected females and one non-infected female control revealed that the parasite targets primarily body wall muscle cells and possibly the adjacent hypodermis; yet parasites are absent from oocytes, developing eggs, and the intestine (Figures 4A–F). Infection often induces the formation of a cyst-like structure comprising intracellular stages (sporonts and sporoblasts) and disorganized tissue, presumably components of the infected cells (Figures 4G; S4; Movie S1). Typically, a nematode body wall muscle cell comprises a contractile region, primarily underlying the cuticle, and an inner non-contractile region. The contractile region includes repeating units of thin and thick filaments that are bundled in a stereotypic organization to generate the mechanical force required for the worm's locomotion. The standard cellular processes are taking place in the non-contractile region where organelles such as nucleus and mitochondria are distributed. Although the number of animals analyzed by TEM was limited by the challenges of sample preparation, the few animals examined exhibited a correlation between the number and distribution of spores and the severity of target cell decomposition. In moderately-infected worms, where spores are confined into a cyst-like structure, muscle decomposition was restricted to the non-contractile part of the body wall muscle (Figure 4E). In more severe cases of infection, where spores are spread in the entire mid-body of the host, decomposition of muscle filaments was observed (Figures 4H,I). Thin cross sections through developing and mature spores revealed their ultrastructural details including two nuclei, a posterior vesicle, and three to five coils of the polar filaments (*n* = 15) (Figure 5).

Collectively, these analyses suggest that *N. marisprofundi* infection starts by invasion through the genitals (i.e., vulva and cloaca), targeting the body wall muscle cells where the parasites complete intracellular phases, mainly in the non-contracting region of the muscle cells (Figure 4E). Spores then translocate to the genital tract for a new infection cycle (Figure 5).

### *Nematocenator* parasites are presumably founding members of a new basal microsporidia clade

**Figure 6:**
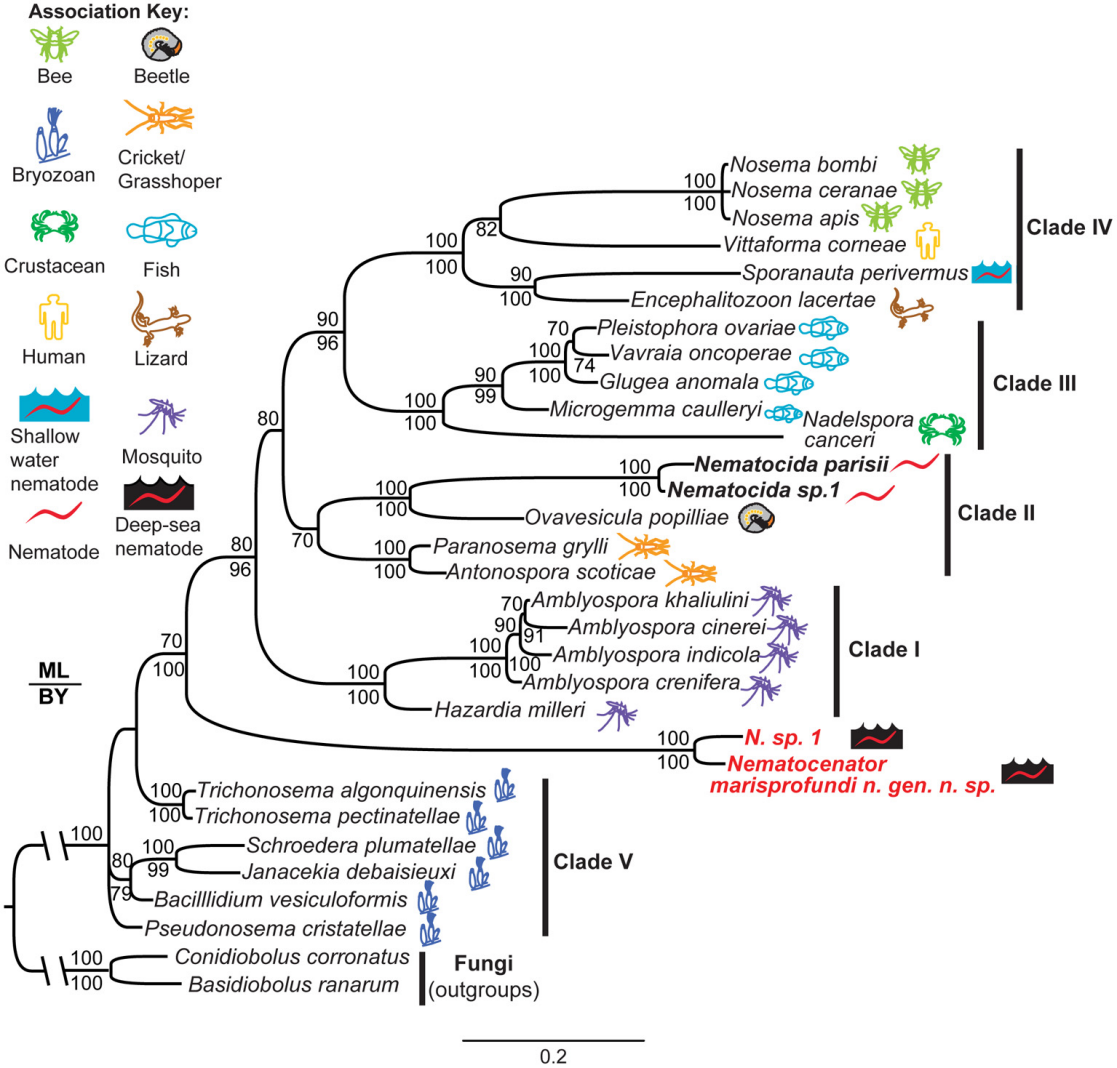
**Phylogenetic analysis of *N. marisprofundi***. A combined maximum likelihood and Bayesian analysis of the small ribosomal DNA from selected microsporidia and close relatives. Two zygomycete fungal taxa were used as outgroups (see Methods). The five major microsporidian clades are noted with Roman numerals (after Vossbrinck and Debrunner-Vossbrinck, 2005). The ecological associations of microsporidian taxa are noted with colored icons. Maximum likelihood bootstrap support values are reported above branch nodes and concordant Bayesian posterior probabilities are reported below each node. Support values <70 were not reported.

To understand the evolutionary origin of *N. marisprofundi*, we have tested phylogenetic relationships to known microsporidia species that occupy marine, fresh water, and terrestrial habitats. Based on molecular data and host association, microsporidia can be divided into five clades (Vossbrinck and Debrunner-Vossbrinck, 2005). Due to the scarcity of *N*. sp. 1's nematode host, we could recover only a portion of its rDNA, which was used to show its close relationship with N. *marisprofundi* (Figure 6). We performed comprehensive phylogenetic analyses with N. *marisprofundi* rDNA and included representative species from all five clades, including the five species that exhibit the highest sequence similarity to *N. marisprofundi* by BLAST analysis (Figure 6). Intriguingly, both maximum likelihood and Baysian analyses concordantly place *N. marisprofundi* as the basal and plausibly founding member of a clade herein interpreted to be at the rank of new genus within microsporidia, not closely related to any other known genera. The *Nematocenator* lineage is predicted to branch at the transition from the most basal group of bryozoan-infecting microsporidia (Canning et al., 2002; Morris et al., 2005) and species that infect other phyla such as nematodes, arthropods, and chordates. The position of *Nematocenator* relative to other nematode-infecting microsporidia (Figure 6; Troemel et al., 2008; Ardila-Garcia and Fast, 2012) demonstrates independent instances of nematode parasitism by microsporidia, with the deep-sea event likely occurring much earlier in microsporidia evolution or with host specialization having occurred late in this ancient microsporidia lineage.

## Discussion

We have discovered a temporally-stable parasitic association of microsporidia and nematodes in deep-sea methane seeps. Our results demonstrate that methane seep ecosystems support not only prokaryotes and animals but also fungal parasitism. To our knowledge, *N. marisprofundi* is the first fungal pathogen shown with its host to be part of deep-sea methane seep ecosystems. Our findings highlight the involvement of a new player, microsporidia, in deep-sea biomass-rich chemoautotrophic food webs.

We tried but were unable to establish a stable host-parasite culture in the lab beyond 6 weeks, where worms were kept at 4°C in semi-dysoxic conditions created by displacing atmospheric air with nitrogen (see Supporting Information for details). Post-mortem analysis revealed that four out of the seven worms in this culture were infected with microsporidia. We could not detect any behavioral or locomotory differences among the nematodes in this culture, but our analysis was necessarily limited. Our ability to keep *N. marisprofundi* infected hosts alive in the laboratory for at least 6 weeks suggests that *N. marisprofundi*-induced disease may be more chronic in nature than the acute pathology induced for example by *N. parisii*, which kills 50% of its *C. elegans* host within 127 h of infection (Troemel et al., 2008). It is possible, therefore, that N *. marisprofundi*'s life cycle inside host cells is significantly slower than that of other microsporidia and that the large number of spores observed in many infected animals is the outcome of many months, or even years of infection. In support of this hypothesis FISH and TEM analyses rarely resulted in detecting developing stages inside infected cells, suggesting that spores are the prominent stage of the *N. marisprofundi* life cycle. The TEM analysis, however, revealed that the parasitic infection induces body-wall muscle degradation by decomposing cellular components including muscle filaments. Therefore, it is likely that *N. marisprofundi* reduces host fitness significantly, at least in severe infection cases (Figure 4H). Because the nematode host is the second most abundant animal species at some HR habitats (Figure 2), microsporidia infection may have gross indirect effects on the HR food web, for example by affecting the abundance of the host, its predators, and its bacterial prey.

Many free-living or phoretic terrestrial nematodes including *C. elegans* are hermaphroditic and isolated from the wild primarily as non-reproducing dauer larvae (Felix and Braendle, 2010). In contrast, HR nematodes are most likely gonochoristic (male-female) and are found in the methane seeps primarily as reproducing adults. The obligate sexual reproduction and abundance of adult stages of the nematode host may shape the transmission strategy of the parasite, constraining it toward vertical or sexual transmission in contrast to the common fecal-oral transmission from environmental spores. A horizontal transmission strategy relying on an oral-fecal cycle would probably have resulted in a low ratio of infecting spores per total number of spores, reducing the fitness of the parasite. Reproduction that requires direct contact between organisms from the same species could be a much more efficient transmission strategy in deep-sea environments such as HR, where food is plentiful and the nematodes are very abundant. Vertical transmission is the strategy of many microsporidia species including the recently characterized microsporidia that infect nematodes at the low tide zone (Ardila-Garcia and Fast, 2012). However, spore distribution in male and female genitals, the conspicuous absence of microsporidia and spores from gonads, eggs and eggshells, in addition to the lack of FISH signal in gonads and developing eggs all support a sexual transmission strategy. We could not test this hypothesis directly by mating-induced infection, due to the non-culturable nature of the samples.

Microsporidia bioenergetics is proposed to rely on the host's ATP and, at least in some cases, on substrate-level phosphorylation (Katinka et al., 2001). Microsporidia are found in many environments including terrestrial niches where oxygen concentration is relatively high. However, a negative correlation between *N. marisprofundi* abundance in Hydrate Ridge and oxygen levels may represent an adaptation of *N. marisprofundi* to anaerobic metabolism advantageous in oxygen-depleted environments such as oxygen minimum zones on continental margins, and in methane seeps and hydrothermal vents. Seep sediments support anaerobic methane oxidation featuring highly dysoxic porewaters whereas in hydrothermal vents gross temperature variations result in very low levels of dissolved oxygen in specific habitats (McMullin et al., 2000). Strikingly, *D. marci* has been found previously in hydrothermal vents in the Lau Basin of the East Pacific Rise (Verschelde et al., 1998), suggesting a wide range distribution of the host nematode in the Pacific Ocean. Moreover, the two sequences most closely related by BLAST search to the host *D. marci* SSU rDNA are of unidentified nematode species recovered from deep-sea hydrothermal vents at the Mid Atlantic Ridge (Lopez-Garcia et al., 2003) (Figure S4). This raises the possibility that along with their hosts, microsporidia parasites are wide spread in chemoautotrophic niches but dispersal of the parasite by spreading of the host over great distances needs further study.

A detailed characterization of eukaryotic host-parasite interactions in the deep sea is currently limited to a few cases such as the mussel-infecting epizootic fungus found in a Fiji Basin hydrothermal vent (Van Dover et al., 2007) and trematode parasitism of mussels at hydrothermal vents and cold seeps (Moreira and Lopez-Garcia, 2003). Our findings present a new perspective on the abundance, nature, and ecological significance of deep-sea parasitism by placing nematodes, one of the most abundant animal phyla in many deep-sea settings, as a host for microsporidia parasites. We propose that parasitism is more widespread in deep-sea environments than previously considered. The apparent paucity of deep-sea parasitism is due not only to limitations in sampling (Jones, 2011), but because some parasites such as microsporidia can be easily overlooked due to extracellular phases that appear bacteria-like. Moreover, deep-sea biodiversity studies rely primarily on the use of degenerate PCR primers limited by design to detect taxa with conserved SSU rDNA sequences. The lack of amplification of *N. marisprofundi* either with degenerate “universal” eukaryotic primers or with fungal-specific ITS primers (data not shown) suggests that broad-scale biodiversity surveys of extreme environments are able to recover only some of the diversity that occurs in these habitats. In addition to primer bias, lack of detection may stem from insufficient lysis of the fungal spores during DNA extraction. The recent discovery of microsporidia-infected nematodes at the lower tide zone (Ardila-Garcia and Fast, 2012) suggests that microsporida parasitism is a characteristic of various marine environments and is presumably much more widespread in the marine realm than previously considered. Microsporidia infections of nematodes at HR methane seeps indicate the potential for unidentified cases of fungal parasitism in deep-sea chemosynthetic environments.

## Materials and methods

### Sample collection and nematode recovery

Samples for nematode analyses were recovered primarily from sulfide oxidizing microbial mats or authigenic carbonates collected from Hydrate Ridge North, Hydrate Ridge South, and East Knoll (see Figure 1 and Table S1 for site coordination) on two research cruises on R/V *Atlantis* in 2010 and 2011 using the HOV Alvin or the ROV Jason II. Upon shipboard recovery all samples were stored at 4°C (close to *in situ* temperature) until further processing (within 2–7 h of recovery). Nematodes were handpicked from washed and sieved (300 μm) samples using forceps under a dissecting microscope (Leica) and stored in cold 4% formaldehyde or 2.5 gluteraldehyde and filtered seawater buffer, or alternatively in a 50:50 ethanol: filtered seawater. Additionally, bulk microbial mat samples, rock animals that were recovered by rock washing and incubation in sea water for 24 h, and crude sediments were collected and stored at 4°C in 50 ml falcon tubes for live sample sorting upon return to California Institute of Technology or Scripps Institution of Oceanography. We examined the potential of parasite spreading in the deep sea by colonization of infected hosts by placing rock, wood, and cow bones next to active seeps and non-active area of Hydrate Ridge North and South for a yearlong colonization experiment starting in August 2010. In September 2011 these substrates were collected from the sites, washed in sea water to recover associated nematodes that were fixed in ethanol or formalin. Next, *D. marci* worms were sorted and examined for the presence of microsporidia infection as described above. For a detailed description of worm and species number per site, and number of worms that were analyzed in each assay see Table S1 and Supporting Information. To establish a dysoxic culture, live worms were individually picked and transferred to glass culture bottles half filled with sea water and sediment from Hydrate Ridge South. The bottles were sealed with rubber caps then flushed with nitrogen to disperse residual atmospheric air and kept at 4°C in the dark and visualized every week for about 3 months. Nematodes viability was determined based on their locomotion and after 6 weeks one bottle containing seven live worms was analyzed for infection percentage.

### Nematode rDNA amplification and sequencing

Small ribosomal subunit 18S (SSU) rDNA of HR nematodes was isolated, amplified, and sequenced as described by Holterman et al. (2006) and in Supporting Information Materials and Methods. Nematode accession numbers in NCBI: *D. marci* JX463180; *Desmodora* sp. 9 JX463181; *Prochaetosoma* sp. 10 JX463182.

### *N. marisprofundi* rDNA amplification and sequencing

Individual non-infected and infected worms were sorted for PCR analysis. A buffer-only PCR was always performed in parallel as a negative control as well as DNA of a non-Desmodorida worm species belonging to Enoplea class isolated from the same site that were not infected by microsporidia. Samples were only used for cloning and sequencing if the negative controls gave no signal by gel electrophoresis. Positive PCR products were cloned into the TOPO TA vector (Invitrogen) and inserts were sequenced using primers that flank the insert of the vector. Detailed information on primers and methods used is in Supporting Information Materials and Methods. A final contig of 1165 bp of *N. marisprofundi* SSU rDNA sequence was reconstituted and submitted to Genebank- accession number JX463178. *Nematocenator* sp. 1 SSU rDNA was recovered using the same methodology but we were able to obtain only 575 bp of high quality sequence that was submitted to Genebank- accession number JX463179.

### FISH reactions of *D.marci* worms

Probes were designed against regions of the ribosomal sequence specific to *N. marisprofundi* and were synthesized with a Quasar 570 (Cy3) 5′ modification and HPLC purified by Biosearch Technologies, Inc. FISH was then performed on infected and non-infected worms essentially as described (Troemel et al., 2008) with some modification describe in Supporting Information Materials and Methods.

### Calcofluor white staining of spores

Calcofluor White (Faluka) staining of infected animals and Calcofluor White-based spore survey of environmental samples collected from Hydrate Ridge is describe in Supporting Information Materials and Methods.

### Morphological nematode study

Nematode individuals from 50:50 ethanol: sea water samples were slowly evaporated to anhydrous glycerol before mounting on glass slides following (Somerfield and Warwick, 1996). Nematodes were identified under a compound microscope with differential interference contrast illumination to species level using pictorial keys (Platt and Warwick, 1988), and the NeMys database (Deprez et al., 2005) with identification keys to the families Desmodoridae and Draconematidae.

### Transmission and scanning electron microscopy

Nematodes for scanning electron microscopy were handpicked, fixed, and critical point dried before imaging. For transmission electron microscopy, crude samples were fixed in 2.5% gluteraldehyde in sea water and stored in fixative solution at 4°C. Before embedding, samples were washed in PBS three times, female *D. marci* worms were picked and mounted on a thin film of 2% agar on slides and were examined for spores by Nomarski DIC microscopy. Detailed transmission and scanning electron microscopy methods are in Supporting Information Materials and Methods.

## Author contributions

Victoria J. Orphan, Lisa A. Levin, Paul W. Sternberg, Adler R. Dillman, and Amir Sapir conceived and design the experiments and analyzed the data. Lisa A. Levin, Benjamin M. Grupe, and Victoria J. Orphan collected, processed, and analyzed the samples shipboard. Amir Sapir, Adler R. Dillman, Stephanie A. Connon, and Benjamin M. Grupe processed, sorted out and analyzed nematodes and other animals from the samples in the lab. Manuel Mundo-Ocampo and James G. Baldwin conducted SEM analysis, Jeroen Ingels, James G. Baldwin, Adler R. Dillman and Amir Sapir conducted worm taxonomy and analysis of worm morphology changes upon infection. Amir Sapir did the FISH reactions, Calcofluor White staining, and the TEM imaging. Adler R. Dillman conducted nematode and microsporidia phylogeny studies and worm diagram drawing. Amir Sapir, Adler R. Dillman, Victoria J. Orphan, Lisa A. Levin, and Paul W. Sternberg wrote the paper.

### Conflict of interest statement

The authors declare that the research was conducted in the absence of any commercial or financial relationships that could be construed as a potential conflict of interest.
