# Supplementary material for: Microsporidia-nematode associations in methane seeps reveal basal fungal parasitism in the deep sea
Source: Front Microbiol. 2014 Feb 10;5:43. doi: 10.3389/fmicb.2014.00043 (PMC3918590; doi:10.3389/fmicb.2014.00043)
Supplement: Supplementary file 3 [file Presentation1.PDF]

Table S1. Microsporidia-infected *D. marci* distribution at Hydrate Ridge carbonate rocks and colonization experiments

Cruise/ year/date	DIVE/ elevator/ site	Coordinates X,Y lat, long	depth	Site description	Infected/ non infected *D. marci* worms	Total infection percentage
AT-18-10 09-06-2011	E11a HR12	(1076; 1973) 44 34.0653N 125 9.1876W	797 m	Hydrate Ridge South, off Pinnacle Inactive carbonate rock partially sedimented	No *D. marci* worms	NR
AT-18-10 09-06-2011	E11a, Box 12	(1519; 2213) (44 34.1949 N, 125 8.8531 W)	775 m	Hydrate Ridge South, Mounds Area, carbonate on sediment with some mat no infected worms	No *D. marci* worms	NR
AT-18-10 09-04-2011	E7a HR9	(1113; 2049) 44 34.10797 N 125 9.16943 W	774 m	Hydrate Ridge South, Pinnacle active site	24 males & 25 females/ 25 males & 6 females*	61.25%
AT-18-10 09-04-2011	E7a HR9	(1100, 2052) 44 34.10797 N, 125 9.16943 W	774 m	Hydrate Ridge South, Pinnacle Sterile carbonate active site incubation sample HR9-I	2 females& 3 males / 2 females & 1 male**	62.50%
AT-18-10 09-04-2011	E7a HR9	(1102; 2048) 44 34.10797 N 125 9.16943 W	774 m	Hydrate Ridge South, Pinnacle active carbonate loose in sediment Sedimented rock 2	3 males & 5 females/ 6 males & 4 females	44.40%
AT-18-10 09-03-2011	E7a HR9	(1113; 2049) 44 34.10797 N 125 9.16943 W	774 m	Hydrate Ridge South, Pinnacle- active carbonate with microbial mat	3 females/ 7 females & 1 male	27.27%
AT-18-10 09-01-2011	E1a HR3	44 40.17212 N 125 9.16943 W	588 m	Hydrate Ridge North (near HR3) Carbonate from 'inactive' site	No *D. marci* worms	NR
AT-18-10 09-01-2011	E1	(5447, 13283) 44 40.17212 N, 125 5.88719 W	587 m	Hydrate Ridge North, NF-3 HR-3 wood colonization	1 male non infected	NR
AT-18-10 09-01-2011	E1a HR3	44 40.17212 N 125 9.16943 W	588 m	Hydrate Ridge North (at HR3) carbonate from 'active' site – very sulfidic, lots of animals colonizing carbonate	No *D. marci* worms	NR
AT-18-10 09-01-2011	E2b HR4	(5403; 13343) 44 40.2045N 125 5.9204W	595 m	Hydrate Ridge North (at HR4) Natural fir wood at inactive site in situ incubation for 13 months	No *D. marci* worms	NR
AT-18-10 09-01-2011	E1a HR3	(5442, 13269) 44 40.1646N 125 5.8910W	587 m	Hydrate Ridge North (HR3) Inactive site carbonate with foraminifera	No *D. marci* worms	NR
AT-18-10 09-04-2011	E8b	(1561; 2249) 44 34.2143N 125 8.8214W	775 m	Hydrate Ridge South, Carbonate rock on sediment and mat (near HV1)	No *D. marci* worms	NR
AT-15-68 08-07-2010	AD4635 HR11	(1077; 1989) 44 34.06640 N 125 9.19662 W	795 m	Hydrate Ridge South, Pinnacle, ledge white microbial mat Sponge rock S7; active site	No *D. marci* worms	NR
AT-15-68 08-07-2010	AD4635 HR9	(1103; 2047) 44 34.10797 N 125 9.16943 W	774 m	Hydrate Ridge South, Pinnacle, carbonate rock E4	16 males & 37 females / 24 males & 29 females***	50%
AT-15-68 08-05-2010	AD4633	(10988; 11375) 44º 26.99N; 125º 01.97W	625 m	South East Knoll, Orange mat Thioploca sp. collected with scoop	1 male & 1 female/ 1 male &1 female	50%
AT-15-68 08-05-2010	AD4633	(10987; 11376) 44 26.85810 N, 125 1.70340 W)	625 m	Hydrate Ridge South east knoll, Rock S5	No *D. marci* worms	NR
AT-15-68 08-04-2010	AD4632	(5473; 13229) 44 40.14296 N, 125 5.86756 W	591 m	Hydrate Ridge North Rock, E3B- active site	2 females non infected	NR
AT-15-68 08-04-2010	AD4632	(5473, 13229) 44 40.14296 N, 125 5.867856 W	591m	Hydrate Ridge North, E2 rock from HR-3 with orange bacteria	134 *D. marci* worms - all non-infected	NR
AT-18-10 08-02-2010	AD4630	(5447; 13274) 44 40.16726 N, 125 5.88719 W	587 m	Hydrate Ridge North, Rock S8 active site	No *D. marci*	NR
AT-15-68 08-01-2010	AD 4629 HR1	(1120; 2097) 44 34.11067 N 125 9.15886 W	776 m	Hydrate Ridge South Pinnacle 'Santa's ledge' Carbonate 'jefe' rock E4; active site Shipboard incubation experiment	2 males / 4 males	33.30%

Colonization experiments 2010-2011 (dates corresponding to substrates placement time). All colonization experiments were recovered on September 2011

AT-18-10 08-03-2011	E5a	(4613; 13206) 44 40.13054 N, 125 6.51691 W	615 m	Hydrate Ridge North, WG HR-6 Wood substrate	2 males non infected	NR
AT-18-10 08-03-2011	E5a	(4613; 13206) 44 40.13054 N, 125 6.51691 W	615 m	Hydrate Ridge North, RG HR-6 Rock substrate	4 females non-infected & 3 non-infected males	NR
AT-18-10 08-03-2010	E5a	(4613; 13206) 44 40.13054 N, 125 6.51691 W	615 m	Hydrate Ridge North, HR-6 Rock substrate F E5A	1 non-infected male	NR
AT-18-10 08-01-2010	HR-1	(1114; 2057) 44 34.11067 N 125 9.15886 W	774 m	Hydrate Ridge South HR-1 Rock 1 substrate	16 females & 3 males/ 5 females & 5 males****	63.33%
AT-18-10 08-01-2010	HR-1	(1114; 2057) 44 34.11067 N 125 9.15886 W	774 m	Hydrate Ridge South HR-1 Bone-1 substrate	3 females & 2 males/ 13 females & 12 males	16.66%
AT-18-10 08-01-2010	HR-1	(1114; 2057) 44 34.11067 N 125 9.15886 W	774 m	Hydrate Ridge South HR-1 Wood-1 substrate	2 females & 1 male/ 13 females & 14 males	10.00%
AT-18-10 08-07-2010	HR-9	(1100; 2052) 44 34.10797 N 125 9.16943 W	775 m	Hydrate Ridge South HR-9 Rock 9 substrate	8 females & 4 males/ 9 females & 9 males	40.00%
AT-18-10 08-08-2010	HR-11	(1064; 1975) 44 34.06640 N 125 9.19662 W	800m	Hydrate Ridge South HR-11 Rock 11 substrate	1 female & 2 males/ 11 females & 11 males	10.00%

Table sorted by date of sampling.
In addition to carbonate rocks, twenty five sediment samples from HR North and South were analyzed and found not to hav *D. marci* nematodes.
*1 female- not sure if infected or not.
**2 infected animals were analyzed a week after sampling, 6 nematodes were alive for about 6 weeks and analyzed after 8 weeks.
***3 females- not sure if infected or not.
****1 male not sure if infected or not.
NR- not relevant.
