# Supplementary material for: Microsporidia-nematode associations in methane seeps reveal basal fungal parasitism in the deep sea
Source: Front Microbiol. 2014 Feb 10;5:43. doi: 10.3389/fmicb.2014.00043 (PMC3918590; doi:10.3389/fmicb.2014.00043)
Supplement: Supplementary file 4 [file DataSheet1.DOCX]

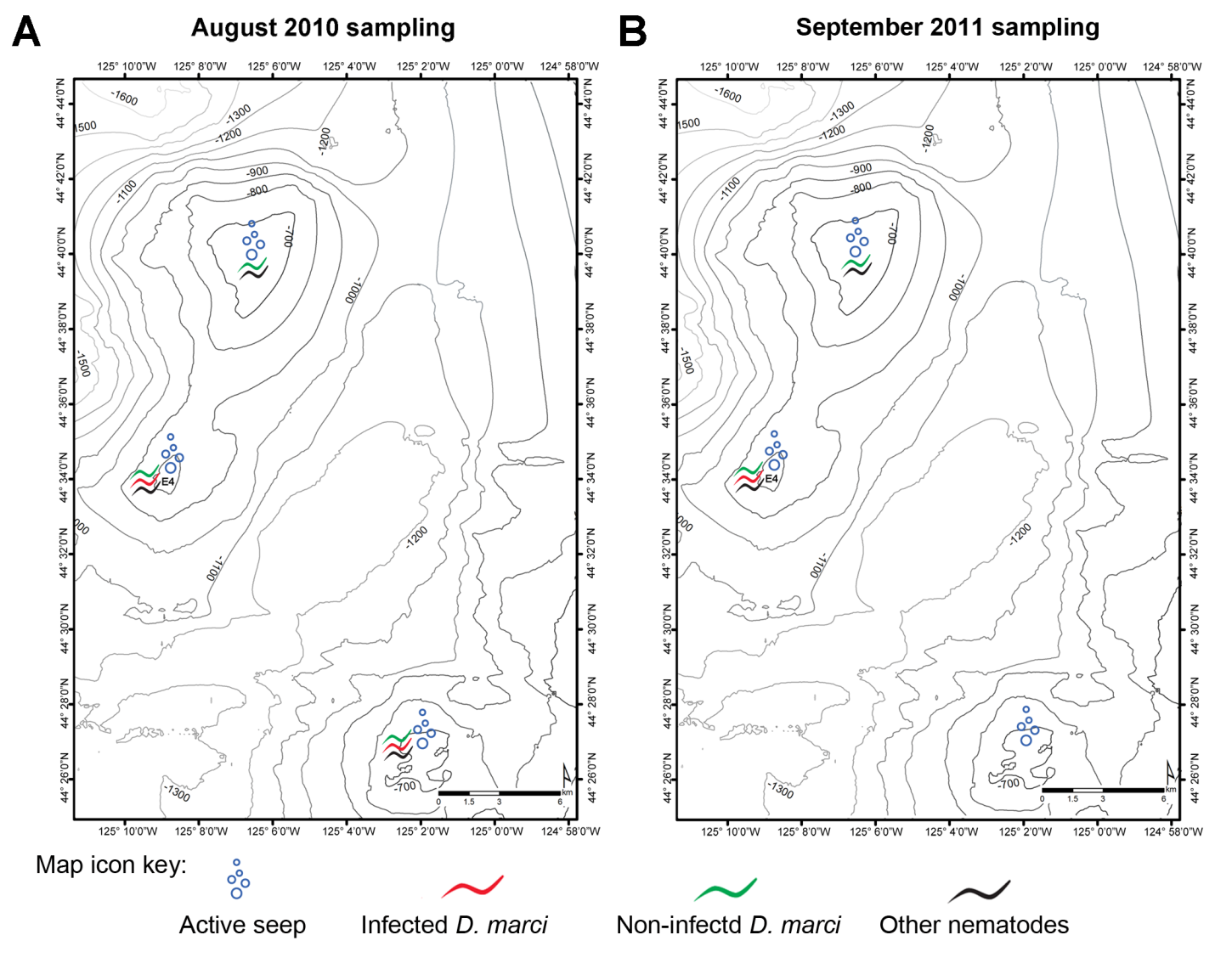


**Fig. S1 *N. marisprofundi* infection is stable at Hydrate Ridge**

(A) August 2010 sampling sites. Infected *D. marci* nematodes have been found at Hydrate Ridge South and East Knoll active seeps but absent from HR North (n=134). The position of E4 rock samples (Fig. 2) at HR is labeled as E4. (B) September 2011 sampling sites exhibit an infection pattern similar to 2010: Infected *D. marci* at HR South versus the dwelling of non-infected *D. marci* nematodes at HR North (n=7). East Knoll was not examined in 2011. The distribution of twenty-seven sediments that had been sampled from various locations at HR and surveyed for the presence of *D. marci* nematodes are not presented because these samples did not have *D. marci* worms.





**Fig. S2 Two *Desmodora* and one *Prochaetosoma* species dwell at Hydrate Ridge**

Females (left), males (right), and inserts of the reproductive organs of HR *Desmodora* and *Prochaetosoma* nematodes. (A) *D. marci* female. (B) *D. marci* male characterized by spicules with complex distal capitulum and broad velum (C) *Desmodora* sp. 9 female characterized by having typically only two embryos *in uterus* (D) *Desmodora* sp. 9 male characterized by spicules with simple distal capitulum and broad velum (E) *Prochaetosoma* sp. 10 female fixed while laying an embryo (see insert). *Nematocenator* sp. 1 spores are distributed in the uterus (arrowhead). (F) *Prochaetosoma* sp. 10 male with *Nematocenator* sp. 1 spores near its spicule (arrow). Scale bar for A-F 500 μm; 20 μm inserts (A-D; E-F same magnifications).


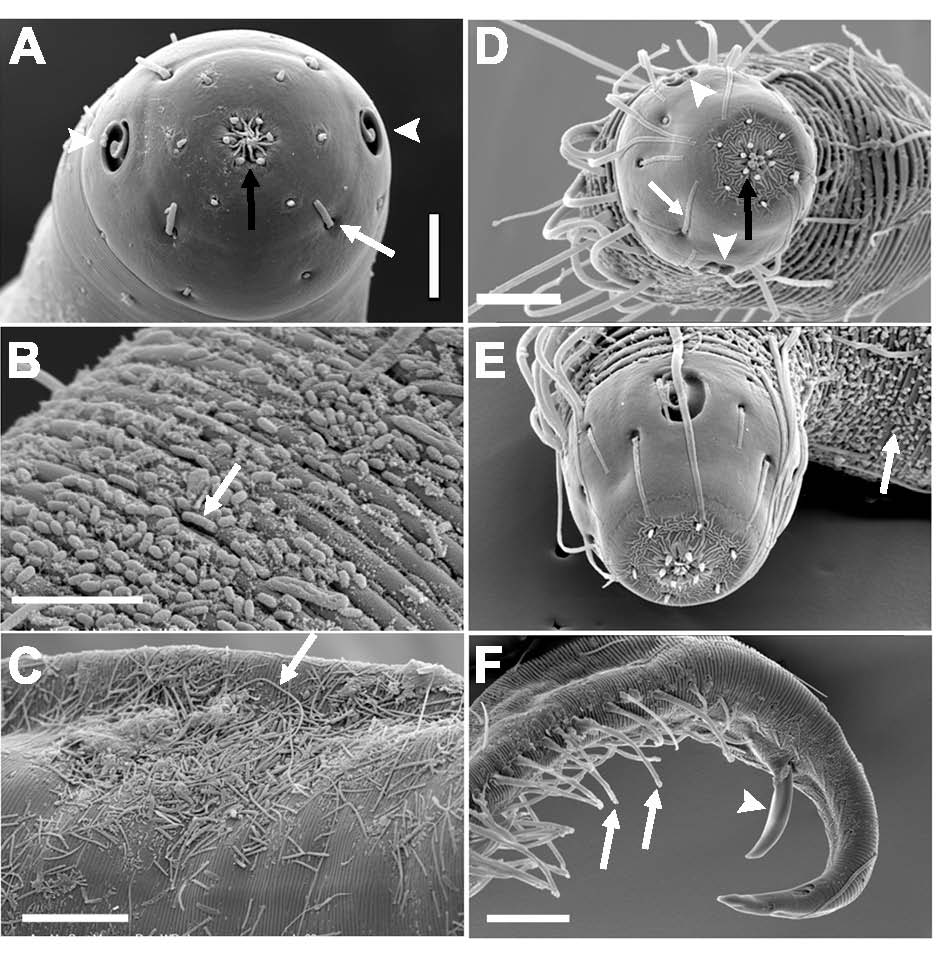


**Fig. S3 Morphological characteristics *of D. marci* and *Prochaetosoma* sp. 10 nematodes**

(A) SEM micrograph of *D. marci* head with head capsule and two cryptospiral amphid sensory organs (1.2 turns, arrowheads), two circles of six labial sensory sensila (setae), four cephalic setae located at a level just in front of the anterior edge of the amphids (one of the four is labeled by a white arrow), and a mouth opening (black arrow). (B) Rod-shaped microorganisms (arrow) are living between the folds of *D. marci* cuticle. (C) Filamentous microorganisms (arrow) are associated with the cuticle of *D. marci* nematodes. (D) SEM micrograph of *Prochaetosoma* sp. 10 head. Open spiral amphid sensory organs (arrowheads), cephalic setae (one is labeled by a white arrow), and a mouth opening (black arrowhead). (E) Rod-shaped microorganisms (arrow) are associated with *Prochaetosoma* sp. 10 cuticle folds. (F) A posterior view of *Prochaetosoma* sp. 10 male with typical subventral and sublateral rows of posterior adhesion tubes (arrows) and a spicule (arrowhead). Scale bars A, D, E, 10 μm; B, 5 μm; C, F, 20 μm.


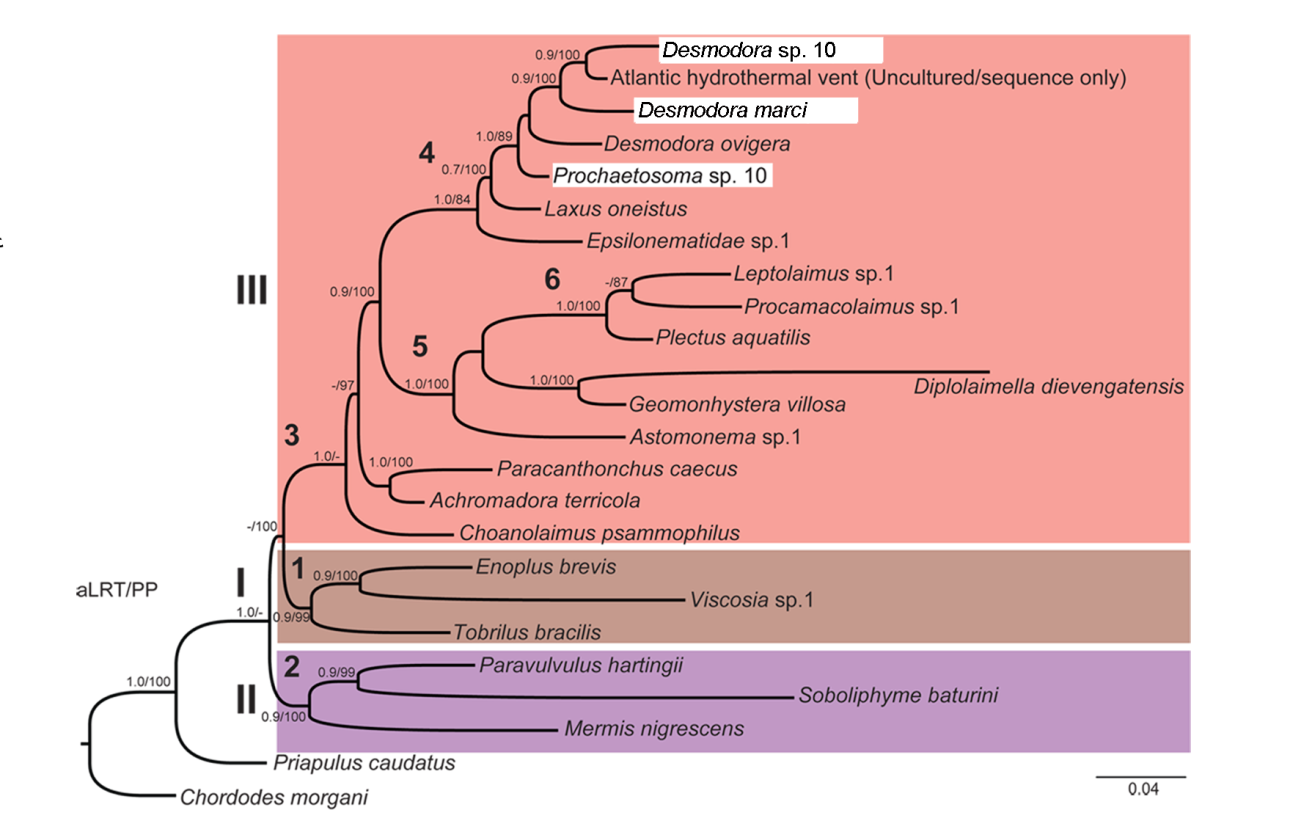


**Fig. S4 Phylogenetic analysis of Hydrate Ridge *Desmodora* and *Prochaetosoma* nematodes**

A combined maximum likelihood and Bayesian analysis of the small ribosomal DNA from selected nematodes and close relatives. A priapulid and a nematomorph are used as outgroup taxa. Nematode clades (1-9) are based on (Holterman et al., 2006) while clades after (Blaxter et al., 1998) are indicated with Roman numerals and colored boxes. At least three taxa from each of the 6 (1-6) Holterman clades are used in this analysis, placing Hydrate Ridge *Desmodora* and *Prochaetosoma* species firmly within clade 4. Atlantic hydrothermal vent worm is the nematode species found to be the most similar to *D. marci* based on BLAST of SSU sequences available in GenBank. This deep-sea worm was collected from sediments at Mid-Atlantic Ridge hydrothermal systems (Lopez-Garcia et al., 2003). *Prochaetosoma* sp. 10 is found similar to Epsilonematidae sp. 1; currently only the families Epsilonematidae and Draconematidae within the superfamily Desmodoroidea are shown to be monophyletic (not Desmodoridae; Decraemer et al., 1997) confirming their close relation. SH-like aLRT and posterior probability support values are placed next to each node (aLRT/PP) where concordant. Support values less than 70 are not reported.


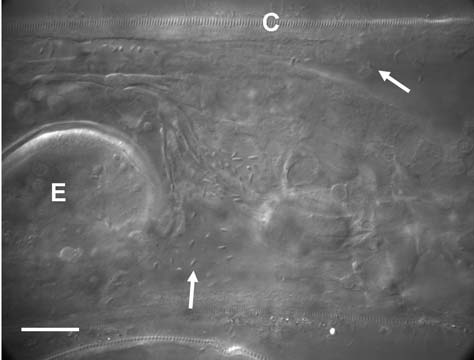


**Fig. S5 *N. marisprofundi* thriving inside live *D. marci* host**

A live worm (see movie S1 for live worm analysis) isolated from Hydrate Ridge South 2011 sample and kept in dysoxic conditions at 4°C for two weeks before analysis. The nematode is infected by microsporidia; E, embryo; C, dorsal cuticle. Arrows point toward spores in the uterus or uterus-adjutant muscles. In a parallel experiment seven *D. marci* animals were kept in dysoxic conditions at 4°C for three months. Three out of the seven found to be infected. Scale bar, 20 μm.


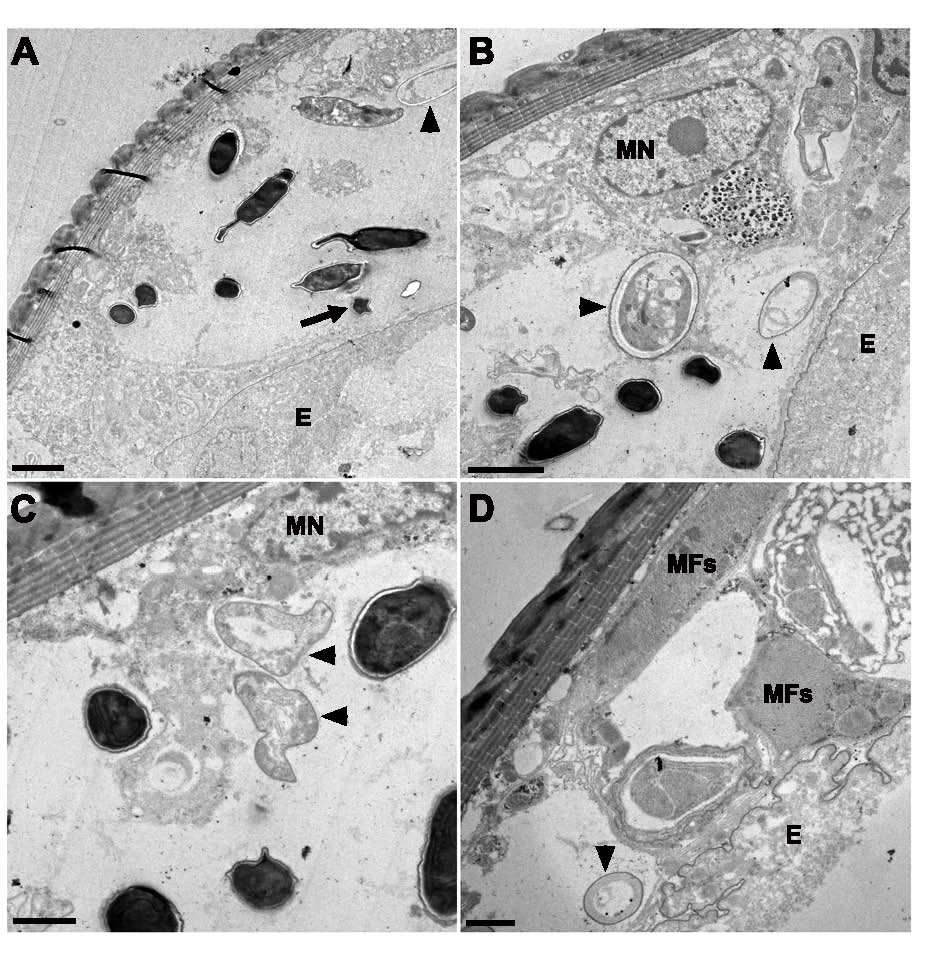


**Fig. S6 Degradation of *D. marci* body wall muscles by *N. marisprofundi***

(A-D) Transmission electron micrographs of 70nm cross sections showing *D. marci* tissue- decomposition by *N. marisprofundi*. E, egg; MN, muscle nucleus; MFs, muscle filaments. (A) Spores and sporoblasts in the remnant of worm tissues. Sporoblast (arrow) and empty spore (arrowhead) (B) Empty (germinated) spores (arrowheads) between an egg and muscle nucleus, scale 2 μm (C) A pair of early sporonts (arrowheads), scale 1 μm. (D) A diplokaryotic sporoblast (arrowhead) adjacent to the muscle filaments. Scale bars A, B, 2 μm; C, D, 1 μm.

**Movie S1 *N. marisprofundi* spores form a cyst-like structure.**

One-micron thick optical Z sections of a *D. marci* female mildly infected with *N. marisprofundi*. Spores are confined in a cyst-like structure on the ventral side of the worm below the gonad. Nuclei are labeled with DAPI. Ventral is down and anterior is to the left. Scale bar 20 μm.

**Movie S2 *N. marisprofundi* thrives on a live *D. marci* host.**

Live *D. marci* female grown in the lab in semi-dysoxic conditions at 4°C and visualized about two weeks after its collection from Hydrate Ridge. Nomarski analysis revealed that the cultured female was infected by microsporidia (Fig. S7).

**Supporting Materials and Methods**

**Physical and chemical condition at the Hydrate Ridge sites**
Site locations:

HR South: 44°34’1’’N 125°9’1” W; HR North: 44° 40’1’’ N 125° 9’1” W; HR South East Knoll 44° 26’8” N 125° 1’7”.

Temperature: 3.2-4.5ºC.
Gas concentrations: based on neighboring CTD casts, O2 concentration is about 0.23 ml/L Hydrate Ridge South and 0.45 ml/L at Hydrate Ridge North.
Pressure: 77.78 atmospheres at Hydrate Ridge South (Rock E4) and 59.1 atmospheres at Hydrate Ridge North (Rock E2).
Salinity: 34.3062 ppt.
light intensity: no light.

Date of sampling: August 2010 and September 2011.

**Number of worms analyzed**

**Detection of infection using light microscopy**

About eight hundred worms from various species were collected and analyzed from HR samples. Five hundred twenty six *D. marci* worms from different locations were analyzed for the presence of spores (Table 1S). For *Procaetosoma* sp. 10 we were able to recover and analyze only fourteen worms.

**rDNA analysis**

For nematode taxonomy, seven *D. marci* males and females and four *Prochaetosoma* sp. 10 nematodes were analyzed using rDNA primers. Microsporidia rDNA sequences were recovered from four *D. marci* and two *Prochaetosoma* sp. 10 infected worms.

**FISH**

Five infected and non-infected *D. marci* worm were analyzed by FISH.

**TEM**

Fourteen infected and four non-infected *D. marci* worms were picked for analysis. After sample processing, four worms (one non-infected, two mildly infected, and one severely infected) were analyzed in details. Eighty thin sections of seventy nanometer and about forty think sections of one micron were imaged using TEM and light microscopy respectively.

**SEM**

We imaged two *D. marci* and one *Prochaetosoma* sp. 10 nematodes.

**Nematode rDNA isolation and amplification**

Nematodes were collected and placed, individually, in 0.2 ml polymerase chain reaction (PCR) tubes containing 25 μl of ultra pure water. An equal volume of lysis buffer (0.2 M NaCl, 0.2 M Tris-HCl (pH 8.0), 1% (v/v) β-mercaptoethanol, and 800 μg/ml proteinase-K) was added to the tubes. Nematodes were then lysed and digested by putting the tubes at 65 °C for 2 h, followed by 5 minutes incubation at 100 °C to heat kill the proteinase K. Lysate was then stored at -20 °C until used. SSU rDNA was amplified in two partially overlapping fragments using three universal and one nematode-specific primer (1912R). The nematode-specific primer was used to avoid non-target eukaryotic contamination. The first of the two fragments was amplified using either primer 988F (5’-ctcaaagattaagccatgc-3’) or 1096F (5’-ggtaattctggagctaatac-3’) in combination with the primer 1912R (5’‑tttacggtcagaactaggg-3’). The second fragment was amplified with primers 1813F (5’-ctgcgtgagaggtgaaat-3’) and 2646R **(**5’-gctaccttgttacgactttt-3’). PCR was performed either by directly adding 2 μl of the crude digestion mix as the DNA template or adding 2 μl of a 100 times diluted crude digestion mix to an Apex^TM^ Taq Master mix. The reaction was then run with following cycling parameters: 94 °C for 5 minutes; 5 X (94 °C for 30 s; 45 °C for 30 s; 72 °C for 70 s) followed by 35 X (94 °C for 30 s; 54 °C for 30 s; 72 °C for 70 s.) and 72 °C for 5 minutes to complete the reaction. Gel purified SSU sequence fragments were then submitted to Laragen (Culver City CA, 90232) for sequencing, using the same primers as were used for amplification.

Nematode accession numbers: *D. marci* JX463180; *Desmodora* sp. 9 JX463181; *Prochaetosoma* sp. 10 JX463182

**Nematode phylogeny**

For the nematode phylogeny (Fig. S4), small subunit ribosomal DNA (SSU rDNA) sequences were obtained from GenBank (FJ040494, U88336, AJ966506, AY284774, AF036641, AY277895, FJ040467, AY593940, AF047888, EF591340, Y16919, Y16913, AJ966482, EF591334, DQ408759, EF591323, EF591326, AF036602, AY284683, AF530552, Z38009, and AF036639). A total of 23 nematode species and two outgroup taxa (a priapulid and a nematomorph) were used. Species were chosen to include 3 species from each of Holterman clades 1-6 (Holterman et al., 2006), the two Desmodora and one *Prochaetosoma* species described in this study, and a nematode species most similar to *D. marci* nematode based on BLAST of SSU sequences available in GenBank. These nematodes were collected from sediments at Mid-Atlantic Ridge hydrothermal systems (Lopez-Garcia et al., 2003). Sequences were aligned using ProAlign (Loytynoja and Milinkovitch, 2003) with 1500 Mb of memory allotted, bandwidth set to 1500 with HMM model parameters being estimated from the data. We excluded characters aligned with posterior probability values under 80%, resulting in 1343 informative characters for subsequent analysis. The TIM2+I+G model was selected as the best-fit model of substitution for analyses using the AICc and BIC model selection criteria in the program jModelTest (Guindon and Gascuel, 2003; Posada, 2009). Maximum likelihood and SH-like aLRT support analyses were carried out in PhyML 3.0 (Guindon et al., 2010) using the parameters for substitution rate matrix, proportion of invariable sites, number of substitution categories, and shape distribution parameter determined as the best-fit by jModelTest (Ra(AC) = 1.4169, Rb(AG) = 3.7073, Rc(AT) = 1.4169, Rd(CG) = 1.0000, Re(CT) = 6.3428, Rf(GT) = 1.0000, p-invar = 0.3250, and gamma shape = 0.5440). Base frequencies were estimated empirically and the p-invar parameter was optimized from the data. Bayesian analysis was carried out using MrBayes 3.1.2 (Huelsenbeck and Ronquist, 2001). The number of substitution categories was based on the parameters determined by jModelTest (as above). Other parameters, such as base frequency, relative rates, substitution rate matrix, and proportion of invariant sites were allowed to vary throughout the analysis. The parameters (shape, statefreq, and revmat) were unlinked to allow for more flexibility in searching tree space. Trees were sampled every 1000 generations. The burn-in value was set to 2000 trees. The total number of generations was set to 8 million. Four parallel chains (one cold and three heated) were used. A majority-rule consensus tree was reconstructed after discarding the burn-in. Support values, where topology was concordant between methods, are placed at branch nodes, with SH-like aLRT values above and posterior probability values below. Support values below 70 were not reported.

**Microsporidia rDNA isolation and sequencing**

Individual non-infected and infected worms were placed in PCR tubes containing 4ul with single worm lysis buffer (50mM Kcl, 10mM Tris pH 8.3, 2.5mM MgCl2, 0.45% NP-40 , 0.45% Tween-20, 0.01% Gelatin, 2 mg/ml proteinase K). Worms were freeze-cracked by subjecting the tubes to five liquid nitrogen-room temperature cycles and lysed with a proteinase K treatment at 65°C for 1 h, followed by 15 minutes at 95°C to inactivate the proteinase K. This extract was then diluted 1:50 in water and 1μl were used per each PCR reaction. A buffer-only PCR was always performed in parallel as a negative control as well as DNA of a different worm species isolated from the same site that were not infected by microsporidia. Samples were only used for cloning and sequencing if the negative controls gave no signal by gel electrophoresis. We used 16S bacterial universal primers b8F-ym (5’-agagtttgatymtggctc-3’) and u1492R-(5’-ggytaccttgttacgactt-3’) and the archaeal universal primers a20F-yr (5’-ttccggttgatccygccrg-3’) and a958R-ym (5’-yccggcgttgamtccaatt-3’) to examine a prokaryotic nature of the parasite. Next, we used eukaryotic universal primers Euk1Af (5’-ctggttgatcctgccag-3’) and Euk516r (5’-accagacttgccctcc-3’) (Diez et al., 2001), the fungal ITS specific primers ITS1 (5’-tccgtaggtgaacctgcgg-3’), ITS2 (5’-tcctccgcttattgatatgc-3’) (White, 1990), and the protozoa-universal primers (5’-ctttcgatggtagtgtattggactac-3’) and (5’-tgatccttctgcaggttcacctac-3’) (Karnati et al., 2003). Because amplification with standard microsporidia-specific primers (Troemel et al., 2008) did not resulted in a reproducible amplicon, microsporidia- specific primers were designed based on sequence alignment of 18rDNA sequences from 19 microsporidia sequences (Franzen et al., 2006) Am-282 (5’-gtgccagcanccgcgg-3’) and Am-283 (5’-gggcggtgtgtrcaaagaac-3’) that gave 950 bp amplicon specific to infected worms and found by sequencing to share high sequence similarity with microsoporidia SSU genes. To amplify the 5’ portion of *N. marisprofundi* rDNA SSU gene we design and used Am-303 (5’-caccaggttgattctgcctgac-3’) in combination with AM-297 (5’-ctgtaccattgtagtgcgcg-3’). Positive PCR products were cloned into the TOPO TA vector (Invitrogen) and inserts were sequenced using primers that flank the insert of the TA vector. A final contig of 1165bp of *N. marisprofundi* SSU rDNA was reconstituted and submitted to Genebank- accession number JX463178.

**Calcofluor White staining of spores**

Calcofluor White staining was performed with the following optimizations: Due to the worms’ cuticle high autofluorescence and lack of staining of intact worms (data not shown), we placed worms on a slide in 5μl water drop and dissected out the spores. Once the drop dried, 5μl of Calcofluor White was added to the worm/spores spot followed by a 5μl drop of 10% KOH and imaged with DAPI filter. There was no apparent difference between staining live and paraformaldehyde or gluteraldehyde fixed samples. Staining of *S. cerevisiae* and *E. coli* were used as positive and negative controls respectively. For environmental survey worms were sorted out of gluteraldehyde-fixed samples and were then washed two times in PBS and vacuum filtered through 0.22 μm isopore black filter (Millipore #GTBP02500). The filters were then placed on a slide and stained with 5μl Calcoflour White and 5μl 10% KOH drops that were spread on the filter. As positive control infected worms from the environmental samples were dissected and spores were detected on the filter after staining.

**FISH of infected worms**

Two *N. marisprofundi* -specific probes were tested MSDS-1 (5’-ggcctcacctagttggcata-3’) and MSDS-2 (5’-gcttcggtacagtttcctgc-3’), MSDS-1 gave a morphologically relevant and microsporidia-specific signal. Worms were individually sorted and fixed on the ship in 4% paraformaldehyde in HR sea water and were kept in the fix solution at 4°C until the time of the experiment. After sorting, worms were individually mounted on a 2% agar slide and examined under DIC microscope for the existence or absent of spores. Worms that didn’t harbor even a single spore were considered as non-infected. FISH was then performed on infected/non infected worms essentially as described (Troemel et al., 2008) following the paraformaldehyde fixation based method where the spores largely remain probe impermeable. Briefly, worm tails were cut using two syringe needles to enable probe penetration. Worm were washed two times with PBS + 0.1% Tween 20 and then transferred to hybridization buffer (900 mMNaCl, 20 mM Tris [pH 7.5], 0.01% SDS) containing 5 ng/ml probe and incubated at 46°C overnight. Next, the hybridization solution was replaced two times by 1 ml wash buffer (900 mM NaCl, 20 mM Tris [pH 7.5], 0.01% SDS, 5 mM EDTA) and incubated at 48°C for 1 hour following by 20 minutes incubation in wash buffer containing 5 μg/ml DAPI. Finally worms were washed two times in wash buffer and were mounted for microscopy with Fluoromount-G (Southern Biotech 0100-01). Confocal optical z-sections of the infection region were collected and were projected to form the final images.

**Scanning electron microscopy**

Male and female nematodes were concentrated in a 25 ml glass vial with 12.5 ml distilled water. The vial was immediately filled with 12.5 ml of a 10% formaldehyde buffer (pH 7.0) that was pre-heated to 65 °C, making the final volume 25 ml and the final formaldehyde concentration 5.0%. After 24 hours of primary fixation, nematodes were rinsed several times with a 0.1 M phosphate buffer. The samples were then transferred to a beam capsule for a post-fixation by a 4.0% osmium tetroxide solution for 4 hours. Post-fixed specimens were rinsed several times within a 15 minutes period with a cold 0.1 M phosphate buffer, and then dehydrated through a series of absolute ethanol aqueous dilutions from 20% through 100%. Dehydrated specimens were critical point dried in a Tousimis autosamdri-810 ® critical point drier, (Rockville. MD, USA), mounted on aluminum stubs, coated with a 25 nm layer of gold palladium in a Cressington® 108 Auto sputter coater, and observed with a XL30-FEG Phillips® SEM at 10 Kv scanning electron microscope.

**Transmission electron microscopy**

Hydrate Ridge E4 rock wash environmental samples from 2010 were fixed immediately upon collection in 2.5% gluteraldehyde in sea water and stored in fixative solution at 4°C. Before sorting, samples were washed in PBS three times and *D.marci* female worms were sorted and mounted on a thin film of 2% agar on slides and were examined for spores by Nomarski DIC microscopy. Worms that did not contain a single spore were classified non-infected, whereas worms with spores were ranked based on the spore distribution, i.e.: i) Mildly infected - a single small spore cyst. ii) Moderately infected - a large cyst or many spores but all confined to the uterus area. iii) Heavily infected - many spores/cysts distributed in mid body and also outside the uterus. We analyzed by transmission electron microscopy two moderately infected worms, one heavily infected worm and one non-infected control. A mid piece from each worm containing the uterus and gonad were dissected by trimming the posterior and anterior ends. This “intact gonad tube” was transferred to a microcentrifuge tube and after several rinses in 0.1 M cacodylate buffer, the samples were post-fixed in 1.0% osmium tetroxide in 0.1 M cacodylate buffer for 1 h at room temperature. Next, gonads were rinsed in 0.1 M cacodylate buffer and then in double distilled water and stained with 2.0% aqueous uranyl acetate for 1 hour at room temperature (for lighter staining of mature spores, this first uranyl acetate staining step was sometimes omitted). After rinsing in distilled water, the samples were dehydrated through a graded series of ethanol to 100%, then infiltrated with epoxy. Infiltration was done by sample incubation in a series of mixtures with ratios of 1:3, 1:1, and 3:1 of EPON/ ethanol (one hour each incubation) and agitation in 100% EPON overnight. Samples were then transferred to fresh 100% EPON for several hours and subjected to a vacuum for 1 hour before embedding in fresh EPON and incubation at 60°C for 48hrs. Thick sections (1 μm) and thin sections (70nm) were cut on a Leica UCT® ultramicrotome**.** Sections 1μm thick were collected on a slide, and stained with 1% toluidene-blue in borax buffer and imaged by light microscopy. Ultrathin 70nm sections were collected on 100 mesh copper formvar-carbon coated grids and post stained with 3% uranyl acetate and lead citrate. Images were collected using an FEI Tecnai electron microscope at 120keV equipped with an Gatan 2 x 2K CCD.

| **Microsporidia phylogeny**  Small subunit ribosomal DNA (SSU rDNA) sequences for the Microsporidia phylogenetic analysis were obtained from GenBank for all taxa included in the present study (accession numbers: NG017184, AF296753.1, AF484694, AJ581995, AY135024.1, AJ252950.1, AY582742, AF484695, AY305325.1, AF024655.1, EF564602, FJ005051.1, FJ005052.1, AY090067.1, AY090051, AY090043, AY090045, AY090056, AY958070, AY033054, AF056016.1, AJ278955, X74112, L39112, AF067144, AY008373, X73894, and U26533). A total of 29 microsporidia species and 2 fungal outgroup taxa (*Basidiobolus ranarum* and *Conidiobolus corranatus*) were used in the analyses for Fig. 6. Taxa were chosen to represent the diversity of the 5 previously identified Microsporidia clades, with at least 5 members representing each clade (Vossbrinck and Debrunner-Vossbrinck, 2005; Troemel et al., 2008). Sequences were aligned using ProAlign (Loytynoja and Milinkovitch, 2003) with 1500 Mb of memory allotted, bandwidth set to 1500 with HMM model parameters being estimated from the data. We excluded characters aligned with posterior probability values under 40%, resulting in 980 aligned characters for subsequent analysis. The TIM3+G model was selected as the best-fit model of substitution for all analyses using the AICc model selection criteria in the program jModelTest (Guindon and Gascuel, 2003;Posada, 2009). Maximum likelihood and bootstrap (1000 replicates) analyses were carried out in PhyML 3.0(Guindon et al., 2010) using the parameters for base frequencies, substitution rate matrix, proportion of invariable sites, number of substitution categories, and shape distribution parameter determined as the best-fit by jModelTest (freqA = 0.2800, freqC = 0.1766, freqG = 0.2881, freqT = 0.2553, Ra(AC) = 0.7590, Rb(AG) = 1.9696, Rc(AT) = 1.0000, Rd(CG) = 0.7590, Re(CT) = 3.0864, Rf(GT) = 1.0000, p-inv = 0, and gamma shape = 0.5580). Bayesian analysis was carried out using MrBayes 3.1.2 (Huelsenbeck and Ronquist, 2001). The number of substitution categories and shape was based on the parameters determined by jModelTest (as above). The parameters for base frequencies, relative rates, substitution rate matrix, and proportion of invariant sites were allowed to vary throughout the analysis. The parameters (shape, statefreq, and revmat) were unlinked to allow for more flexibility in searching tree space. Trees were sampled every 1000 generations. The burn-in value was set to 2000 trees. The total number of generations was set to 8 million. Four parallel chains (one cold and three heated) were used. A majority-rule consensus tree was reconstructed after discarding the burn-in. Support values, where topology was concordant between methods, are placed at branch nodes, with ML bootstrap values above and posterior probability values below. Support values below 70 were not reported.For *Nematocenator* phylogeny (Fig. S6), small subunit ribosomal DNA (SSU rDNA) sequences were obtained from GenBank (Accession numbers described in Fig. 4). A total of 8 microspiridia species and a fungal outgroup was used. The *Nematocenator* sequences were sequenced from infected nematodes at hydrate ridge with *N. marisprofundi* coming from *D. marci* and *N.* sp. 1 coming from *Prochaetosoma* sp 10*.* Sequences were aligned using ProAlign (Loytynoja and Milinkovitch, 2003) with 1500 Mb of memory allotted, bandwidth set to 1500 with HMM model parameters being estimated from the data. We excluded characters aligned with posterior probability values under 70%, resulting in 477 informative characters for subsequent analysis. The TIM2+G model was selected as the best-fit model of substitution for analyses using the AICc model selection criteria in the program jModelTest (Guindon and Gascuel, 2003; Posada, 2009). Maximum likelihood and SH-like aLRT support analyses were carried out in PhyML 3.0 (Guindon et al., 2010) using the parameters for substitution rate matrix, proportion of invariable sites, number of substitution categories, and shape distribution parameter determined as the best-fit by jModelTest (Ra(AC) = 1.1012, Rb(AG) = 2.1687, Rc(AT) = 1.1012, Rd(CG) = 1.0000, Re(CT) = 3.7366, Rf(GT) = 1.0000, and gamma shape = 0.8360). Base frequencies were estimated empirically and the p-invar parameter was optimized from the data. Bayesian analysis was carried out using MrBayes 3.1.2 (Huelsenbeck and Ronquist, 2001). The number of substitution categories was based on the parameters determined by jModelTest (as above). Other parameters, such as base frequency, relative rates, substitution rate matrix, and proportion of invariant sites were allowed to vary throughout the analysis. The parameters (shape, statefreq, and revmat) were unlinked to allow for more flexibility in searching tree space. Trees were sampled every 1000 generations. The burn-in value was set to 2000 trees. The total number of generations was set to 8 million. Four parallel chains (one cold and three heated) were used. A majority-rule consensus tree was reconstructed after discarding the burn-in. Support values, where topology was concordant between methods, are placed at branch nodes, with SH-like aLRT values above and posterior probability values below. Support values below 70 were not reported.  **Microsporidia taxonomy**  Based on their phylogenic position, spore morphology, host species, and habitat the two new microsporidia species described in our study are clearly distinct from *Nematocida parisii*, which infect *C. elegans* (Troemel et al., 2008)and *Sporanauta perivermis*, which infect *Odontophora rectangular* (Ardila-Garcia and Fast, 2012).Few other species of microsporida have been described from nematodes (Poinar and Hess, 1986); only *Microsporidium rhabdophilum* has been described with modern ultrastructure methods (but no molecular characterization) from the nematode *Rhabditis myriophila* (Poinar and Hess, 1986). *M.* *rhabdophilum* is characterized by uninucleate spores produced in a sporophorous vesicle and thus can be excluded from further consideration. While additional information on other species of microsporidia from nematodes is clearly needed, the morphological and molecular characterization presented here together with a unique host group and habitat warrants proposal of a new genus and species. The taxa established here are intended for the permanent, public and scientific record.  *Nematocenator n. g.*  *Diagnosis.* All stages in direct contact with the host cell cytoplasm. Merogony not known. Sporonts and spores with diplokaryotic nuclei. Parasites of bacteriovorous nematodes in the superfamily Desmodoroidea, families Desmodoridae and Draconematidae. Sequence of the small subunit rDNA characteristic of the type species.  *Etymology:* The genus name *Nematocenator*, “nematode eater” in Latin refers to the finding that this organism eats tissues of the host nematode.  *Type species. Nematocenator marisprofundin n. sp.*  ***Nematocenator marisprofundi* taxonomy**  *Diagnosis.* With characteristics of the genus.  *Dates of sampling*: August 2010 and September 2011.  *Type Locality:* Hydrate ridge, about 85 kilometers off Oregon shore at the Pacific Ocean floor lat/ lon: 44º 34.09N; 125º 9.14E 580-774 meter below sea levels.  Habitat: Rocks, fine sediment and bacterial mats at Hydrate Ridge methane seep. Physical conditions at the site: pressure 77.78 (atm); Light: No light; O2: 0.2 ml/L; Salinity: 34.3062 ppt; Temperature:4.2°C.  *Type Host:* *D. marci* - a marine nematode species originally described from hydrothermal vent samples in the Lau Basin, East Pacific Rise.  *Site of infection:* Female hosts: vulva and uterus, males hosts: dorsal pseudocoelom, and male reproductive organs- vas deferens near the male gonad.  *Site of intercellular propagation:* body wall muscles and possibly hypodermal tissues.  *Prevalence at sampling points rich in nematodes:* 2010: 56% infected females (n=66), 40% infected males (n=40). 2011: 80.6% infected females (n=31), 49% infected males (n=49)  *Developmental stages:* Early developmental stages not observed. All stages in direct contact with the host cell cytoplasm. Sporonts diplokaryotic, developing spores, and empty spores were observed by FISH and TEM.  *Spores:* Live spores measured by light microscopy: Length: 3.36± 1.07 Width: 1.4 ± 0.78 (n=128). Diplokaryotic spores with a lamellar polaroplast, a large posterior vacuole and an isofilar polar filament with 3-5 coils (n=15). Spore wall composed of an unlayered exospore that is approximately half the thickness of the endospore.  *Etymology:* Specific epithet *marisprofundi* means “of the deep sea” referring to the organism’s habitat.  *Molecular characterization:* The nucleotide sequence of N. *marisprofundi* SSU (1165) has been submitted to GenBank with an accession number JX463178  *Deposition of type specimens:* Two type slides will be deposit with the International Protozoan Type Slide Collection, Smithsonian Institution, Washington, DC (USNM Nos.------). Additional slides and specimens embedded in plastic resin are archived at the Sternberg lab.  ***Nematocenator* sp.1 taxonomy**  *Type species. Nematocenator* sp.1  *Diagnosis.* With characteristics of the genus.  *Dates of sampling*: August 2010 and September 2011.  *Type Locality:* Hydrate ridge, about 85 kilometers off Oregon shore at the Pacific Ocean lat/ lon: 44º 34.09N; 125º 9.14E 580-774 meter below sea levels.  Habitat: Rocks, sand and bacterial mats at the rims of Hydrate Ridge cold methane seep. Physical conditions at the site: pressure 77.78 (atm); Light: No light; O2: 0.2 ml/L; Salinity: 34.3062 ppt; Temperature:4.2°C.  *Type Host:* *Prochaetosoma s*p. 10- an undescribed marine nematode species characterized in this study.  *Site of infection:* Female hosts: vulva and uterus, males hosts: dorsal pseudo-celom, and male reproductive organs- vas deferens near the male gonad.  *Site of intercellular propagation:* not determined.  *Prevalence at sampling points rich in nematodes:* not determined  *Developmental stages:* not determined  *Spores:* not determined  *Molecular characterization:* The nucleotide sequence of N. *marisprofundi* SSU (575 bp) has been submitted to GenBank with an accession number JX463179  *Deposition of type specimens:* Two type slides will be deposit with the International Protozoan Type Slide Collection, Smithsonian Institution, Washington, DC (USNM Nos.------). Additional slides and specimens are archived at the Sternberg lab. |
| --- |

**References**

Ardila-Garcia, A.M., and Fast, N.M. (2012). Microsporidian Infection in a Free-Living Marine Nematode. *Eukaryot Cell*. vol. 11 no. 12 1544-1551

Blaxter, M.L., De Ley, P., Garey, J.R., Liu, L.X., Scheldeman, P., Vierstraete, A., Vanfleteren, J.R., Mackey, L.Y., Dorris, M., Frisse, L.M., Vida, J.T., and Thomas, W.K. (1998). A molecular evolutionary framework for the phylum Nematoda. *Nature* 392, 71-75.

Decraemer, W., Gourbault, N., and Backeljau, T. (1997). Marine nematodes of the family Draconematidae (Nemata): a synthesis with phylogenetic relationships. *Hydrobiologia* 357, 185-202.

Diez, B., Pedros-Alio, C., Marsh, T.L., and Massana, R. (2001). Application of denaturing gradient gel electrophoresis (DGGE) to study the diversity of marine picoeukaryotic assemblages and comparison of DGGE with other molecular techniques. *Applied and Environmental Microbiology* 67, 2942-2951.

Franzen, C., Nassonova, F.S., Scholmerich, J., and Issi, I.V. (2006). Transfer of the members of the genus Brachiola (Microsporidia) to the genus Anncaliia based on ultrastructural and molecular data. *Journal of Eukaryotic Microbiology* 53, 26-35.

Guindon, S., Dufayard, J.F., Lefort, V., Anisimova, M., Hordijk, W., and Gascuel, O. (2010). New Algorithms and Methods to Estimate Maximum-Likelihood Phylogenies: Assessing the Performance of PhyML 3.0. *Systematic Biology* 59, 307-321.

Guindon, S., and Gascuel, O. (2003). A simple, fast, and accurate algorithm to estimate large phylogenies by maximum likelihood. *Systematic Biology* 52, 696-704.

Holterman, M., Van Der Wurff, A., Van Den Elsen, S., Van Megen, H., Bongers, T., Holovachov, O., Bakker, J., and Helder, J. (2006). Phylum-wide analysis of SSU rDNA reveals deep phylogenetic relationships among nematodes and accelerated evolution toward crown clades. *Molecular Biology and Evolution* 23, 1792-1800.

Huelsenbeck, J.P., and Ronquist, F. (2001). MRBAYES: Bayesian inference of phylogenetic trees. *Bioinformatics* 17, 754-755.

Karnati, S.K.R., Yu, Z., Sylvester, J.T., Dehority, B.A., Morrison, M., and Firkins, J.L. (2003). Technical note: Specific PCR amplification of protozoal 18S rDNA sequences from DNA extracted from ruminal samples of cows. *Journal of Animal Science* 81, 812-815.

Lopez-Garcia, P., Philippe, H., Gail, F., and Moreira, D. (2003). Autochthonous eukaryotic diversity in hydrothermal sediment and experimental microcolonizers at the Mid-Atlantic Ridge. *Proceedings of the National Academy of Sciences of the United States of America* 100, 697-702.

Loytynoja, A., and Milinkovitch, M.C. (2003). A hidden Markov model for progressive multiple alignment. *Bioinformatics* 19, 1505-1513.

Poinar, J., G. O. , and Hess, R. (1986). Microspon'diuna rhabdophilum sp. n. (Microsporida : Pansporoblastina), a parasite of the nematode, Rlzabditis myriophila (Rhabditina :

Rhabditidae). *Revue Nématol* 9, 369-375.

Posada, D. (2009). Selection of models of DNA evolution with jModelTest. *Methods Mol Biol* 537, 93-112.

Troemel, E.R., Felix, M.A., Whiteman, N.K., Barriere, A., and Ausubel, F.M. (2008). Microsporidia are natural intracellular parasites of the nematode Caenorhabditis elegans. *PLoS Biol* 6, 2736-2752.

Vossbrinck, C.R., and Debrunner-Vossbrinck, B.A. (2005). Molecular phylogeny of the Microsporidia: ecological, ultrastructural and taxonomic considerations. *Folia Parasitol (Praha)* 52, 131-142; discussion 130.

White, T.J.B., T. Lee, S. Taylor, J.W. (1990). Amplification and

direct sequencing of fungal ribosomal RNA genes for phylogenetics. *PCR Protocols: A Guide to Methods and Applications, eds. Innis, M. A., D. H. Gelfand, J. J. Sninsky, and T. J. White. Academic Press, Inc., New York.*, 315-322.
